# Constrained evolution of overlapping genes in viral host adaptation: Acquisition of glycosylation motifs in hepadnaviral precore/core genes

**DOI:** 10.1371/journal.ppat.1010739

**Published:** 2022-07-28

**Authors:** Xupeng Hong, Stephan Menne, Jianming Hu

**Affiliations:** 1 Department of Microbiology and Immunology, The Pennsylvania State University College of Medicine, Hershey, Pennsylvania, United States of America; 2 Department of Microbiology and Immunology, Georgetown University Medical Center, Washington, District of Columbia, United States of America; University of Pittsburgh School of Medicine, UNITED STATES

## Abstract

Hepadnaviruses use extensively overlapping genes to expand their coding capacity, especially the precore/core genes encode the precore and core proteins with mostly identical sequences but distinct functions. The precore protein of the woodchuck hepatitis virus (WHV) is N-glycosylated, in contrast to the precore of the human hepatitis B virus (HBV) that lacks N-glycosylation. To explore the roles of the N-linked glycosylation sites in precore and core functions, we substituted T77 and T92 in the WHV precore/core N-glycosylation motifs (^75^NIT^77^ and ^90^NDT^92^) with the corresponding HBV residues (E77 and N92) to eliminate the sequons. Conversely, these N-glycosylation sequons were introduced into the HBV precore/core gene by E77T and N92T substitutions. We found that N-glycosylation increased the levels of secreted precore gene products from both HBV and WHV. However, the HBV core (HBc) protein carrying the E77T substitution was defective in supporting virion secretion, and during infection, the HBc E77T and N92T substitutions impaired the formation of the covalently closed circular DNA (cccDNA), the critical viral DNA molecule responsible for establishing and maintaining infection. In cross-species complementation assays, both HBc and WHV core (WHc) proteins supported all steps of intracellular replication of the heterologous virus while WHc, with or without the N-glycosylation sequons, failed to interact with HBV envelope proteins for virion secretion. Interestingly, WHc supported more efficiently intracellular cccDNA amplification than HBc in the context of either HBV or WHV. These findings reveal novel determinants of precore secretion and core functions and illustrate strong constraints during viral host adaptation resulting from their compact genome and extensive use of overlapping genes.

## Author summary

Hepadnaviruses infect a wide range of hosts. The human hepatitis B virus (HBV) and woodchuck hepatitis virus (WHV) are two closely related hepadnaviruses. In contrast to the WHV precore protein, which is N-glycosylated, the HBV precore protein lacks N-glycosylation. As precore and core proteins expressed from the overlapping precore/core genes share most of their sequences but have distinct functions, we investigated the roles of the N-linked glycosylation sequons in HBV and WHV precore/core genes. Our results revealed an important role of the N-linked glycosylation sequons in enhancing precore secretion levels and regulating core protein functions in virion secretion and infection. Furthermore, cross-species complementation assays using HBV and WHV core proteins and HBV or WHV genomes defective in core protein expression indicated that both HBV and WHV core proteins could support intracellular viral replication but not virion secretion of the heterologous virus. These results provide novel insights into the evolution of overlapping genes during host adaptation of hepadnaviruses.

## Introduction

The family *Hepadnaviridae* includes five genera, *Orthohepadnavirus*, *Avihepadnavirus*, *Parahepadnavirus*, *Metahepadnavirus*, and *Herpetohepadnavirus*, which infect a wide range of hosts including mammals, birds, fish, frogs, and reptiles [1]. Hepadnaviruses carry a small (ca. 3.2 kb), partially double-stranded, relaxed circular DNA (rcDNA) genome and replicate it through an RNA intermediate, the pregenomic RNA (pgRNA), by reverse transcription [2]. Human hepatitis B virus (HBV), a prototypic member of *Orthohepadnavirus*, causes chronic infection, which afflicts approximately 260 million individuals worldwide and relates to the developments of liver fibrosis, cirrhosis, and hepatocellular carcinoma (HCC) [3]. Woodchuck hepatitis virus (WHV), another member of *Orthohepadnavirus*, is closely related to HBV and naturally infects the Eastern woodchuck (*Marmota monax*) [4]. Because WHV infection in woodchucks closely resembles HBV infection in humans, with the development of either acute or chronic hepatitis and HCC [5], the WHV-infected woodchuck is a valuable animal model for studying HBV pathogenesis and the development of new anti-HBV compounds [5,6].

HBV exploits the human sodium taurocholate cotransporting polypeptide (huNTCP) as its entry receptor [7]. After entry, HBV rcDNA is converted into the covalently closed circular DNA (cccDNA) in the nucleus of infected hepatocytes. HBV utilizes the host RNA polymerase II machinery to transcribe all viral genomic (i.e., pgRNA) and subgenomic RNAs from the cccDNA [8]. Both HBV and WHV share four open reading frames (ORFs): the precore/core genes encode the precore and core proteins, the polymerase gene encodes the reverse transcriptase (RT), the PreS1/PreS2/S gene encodes the three viral envelope proteins (L, M, and S), and the X gene encodes the multifunctional X protein [9]. The 3.5 kb pgRNA is the template for reverse transcription to make the progeny viral DNA genome but also the template for producing viral RT and core protein. The RT and pgRNA together are packaged into a cytoplasmic nucleocapsid (NC) formed by core proteins, where the pgRNA is reversely transcribed to form firstly single-stranded DNA (ssDNA) and then rcDNA by RT [2]. NCs containing rcDNA are considered as mature NCs, which are either secreted as complete virions after coating by viral envelope proteins or recycled back to the nucleus to replenish the cccDNA pool via the intracellular amplification pathway [10–12]. In addition to mature NCs, empty capsids (i.e., without viral genomes) are also directed to envelopment and secreted as empty virions [13–16]. The cccDNA converted from either extracellular infectious virions during infection or cytoplasmic NCs via the intracellular amplification pathway is the molecular basis contributing to the establishment and persistence of HBV infection [17].

The precore/core ORF contains two in-frame initiation codons that encode precore and core proteins using the overlapping sequences. Precore protein is translated from the 3.5 kb precore mRNA using the first initiation codon, while core protein is translated from the second initiation codon on the pgRNA. HBV core protein (HBc) or WHV core protein (WHc) contains an N-terminal domain (NTD) and a C-terminal domain (CTD) connected by a linker [18], and 120 copies of HBc or WHc dimers form the T = 4 icosahedral HBV or WHV capsid [19]. In contrast to HBc or WHc, the precore protein is dispensable for viral replication but is thought to have immunomodulatory properties [20–24]; however, the function of HBV precore protein is poorly characterized due to the lack of suitable, HBV-susceptible animal models. HBV precore protein, the precursor of the secretory HBV e antigen (HBeAg), and the recently characterized PreC antigen, has a 29 amino acids (aa) extension at the N-terminus, which includes a signal peptide sequence that directs precore into the secretory pathway [16,25]. Early studies on WHV indicated that the precore protein is not necessary for viral replication but is critical for viral persistence [26,27]. We recently found that WHV e antigen (WHeAg) is N-glycosylated in the serum of WHV-infected woodchucks or in the culture supernatant of WHV precore-expressing woodchuck hepatoma cells [28], as also reported when it was expressed ectopically in human HEK293 cells [29]. N-linked glycosylation, occurring on the Asn of the NXT/S motif (sequon) (X stands for any aa except Pro) in the endoplasmic reticulum, is known to play important roles in the proper folding of viral proteins, shielding of immunodominant epitopes from recognition, modulation of receptor binding and cell fusion, etc. [30]. It has been reported that the e antigen of duck hepatitis B virus (DHBV), the prototypic member of *Avihepadnavirus*, is also N-glycosylated [31]. Unlike DHBV and WHV, HBV precore gene products are not glycosylated. Since N-glycosylation sites could have been added or lost during viral evolution, any mutations on the precore protein that are located within the precore/core overlapping region would also affect the core protein sequence and its functions, e.g., capsid assembly, pgRNA packaging, cccDNA formation, and virion morphogenesis [32]. Thus, investigating the roles of N-glycosylation sites on WHV and HBV precore/core proteins may help to understand the absence of N-glycosylation sites on the HBV precore/core proteins during evolution.

**Fig 1:**
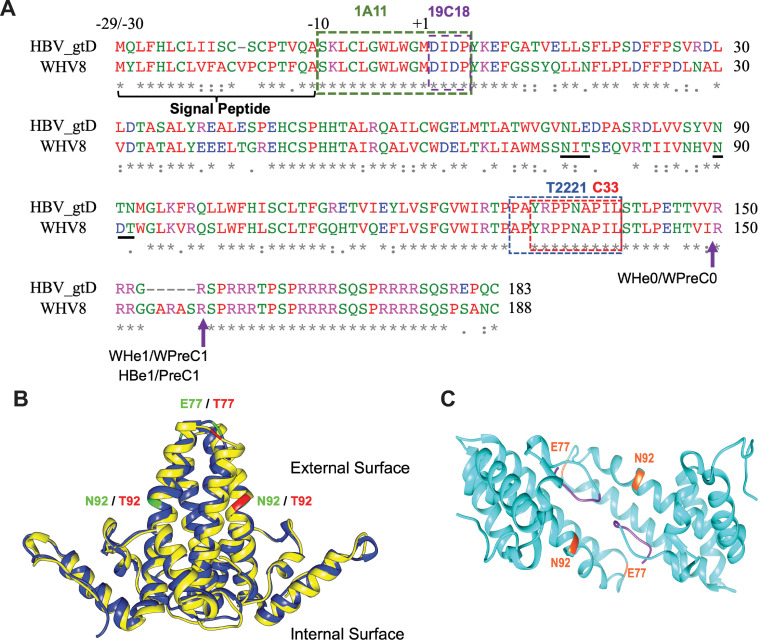
WHV, but not HBV, precore/core gene products are N-glycosylated. **(A)** Amino acid sequence alignment of HBV (genotype D) and WHV (strain 8) precore/core gene products. The epitopes for mAb 1A11, 19C18, T2221, and C33 are indicated by the dashed boxes. The two putative N-glycosylation sequons ^75^NIT^77^ and ^90^NDT^92^ in the WHV precore/core gene are underlined. The arrows indicate the putative cleavage sites in the production of WHeAg (WHe0 and WHe1), WHV PreC protein (WPreC0 and WPreC1), and HBeAg and HBV PreC (HBe1 and PreC1) as identified in a previous study [28]. **(B)** Alignment of the HBc (Protein Data Bank [PDB] accession code 1QGT, blue) and WHc (PDB accession code 6EDJ, yellow) dimer revealed that the two core proteins share highly structural similarity. The T = 4 icosahedral HBV or WHV capsid contains 120 copies of HBc or WHc dimers. Residues E77 and N92 on HBc (in green) and T77 and T92 on WHc (in red) located on the exterior surface of the capsid, which were mutated reciprocally, are highlighted. **(C)** Structure of HBeAg (PDB accession code 3V6Z). The N-terminal extensions are labeled in purple, and the two residues, E77 and N92, which are located on the protein surface, are highlighted.

The WHV genome shares 62 to 70% nucleotide sequence identity with HBV. WHc and HBc share 68% homology in their aa sequences (**Fig 1A**), and a recent comparative analysis of the WHc showed that it is structurally similar to HBc (**Fig 1B**) [19]. A previous *trans*-complementation analysis indicated that either WHV or HBV RT could recognize both HBV and WHV pgRNA packaging signals and form heterologous RT-pgRNA complexes that could be encapsidated into the capsids formed by HBc or WHc [33–36]. We recently showed that either human or woodchuck hepatoma cells are competent to support both WHV and HBV capsid formation, pgRNA packaging, viral DNA synthesis, and cccDNA formation [37]. However, WHV virion secretion is undetectable in these cell culture systems [37,38]. On the other hand, a short linear sequence in the PreS1 domain, the so-called matrix domain (MD), which is thought to interact with the NCs for virion morphogenesis, is conserved between HBV and WHV [39]. Interestingly, the substitution of the HBV PreS1/PreS2 sequence by the WHV PreS1/PreS2 sequence supports HBV virion formation and secretion, albeit inefficiently, in contrast to DHBV [40]. While this result suggests that HBV NCs can partially interact with WHV envelope proteins, it is unknown if WHV NCs can also interact with the HBV envelope proteins to support virion secretion. Furthermore, it was estimated that 20 to 60 copies of WHV cccDNA are present per WHV-infected woodchuck hepatocyte [41], whereas only 1 to 10 copies of HBV cccDNA are present per HBV-infected hepatocyte in humans or chimpanzees [42,43]. The mechanism behind such difference in the intrahepatic cccDNA copy numbers of the two viruses remains unclear.

In this study, we aimed to investigate the roles of N-glycosylation sequons in hepadnaviral precore/core genes. By utilizing mutagenesis analysis, we studied the effects on precore secretion and core protein functions in the HBV and WHV lifecycle in the presence or absence of N-linked glycosylation sequons in the precore/core genes. Moreover, we performed cross-species complementation assays of HBc and WHc with HBV or WHV genomes that are defective for core protein expression to comprehensively investigate the effects of WHc or HBc on supporting intracellular replication steps and virion secretion of heterologous viruses. Our results revealed novel determinants of precore secretion levels and core protein functions, which further provided important insights into hepadnaviral evolution.

## Results

### Primate hepadnaviruses including HBV lack N-linked glycosylation sites in the precore/core genes

We recently found that the secretory WHV precore products are N-glycosylated [28], and another earlier study reported that DHBV e antigen is also N-glycosylated [31]. Among the *Hepadnaviridae*, only avihepadnaviruses and orthohepadnaviruses are expected to express precore proteins and secrete e antigens [44]. We inspected all available hepadnaviral sequences in the database for the N-glycosylation motif, NXT/S, in their precore/core genes. We found that the precore/core genes of all avian hepadnaviruses possess one or two putative N-glycosylation sites (**S1A and S1B Fig**), which has been experimentally verified in the case of DHBV [31]. Conversely, for Orthohepadnaviruses, only WHV, arctic squirrel hepatitis virus (ASHV) [45], ground squirrel hepatitis virus (GSHV) [46], domestic cat HBV (DCHBV) [47], Maxwells’ duiker HBV (MDHBV) [48], and the recently identified equid HBV (EqHBV) [49] have one or two putative N-glycosylation motifs in their precore/core genes (**S1A** and **S1C Fig**), whereas all Orthohepadnaviruses that infect primates or bats lack these N-glycosylation motifs in their precore/core genes (**S1A Fig**).

### N-glycosylation enhanced the levels of secreted precore gene products

**Fig 2:**
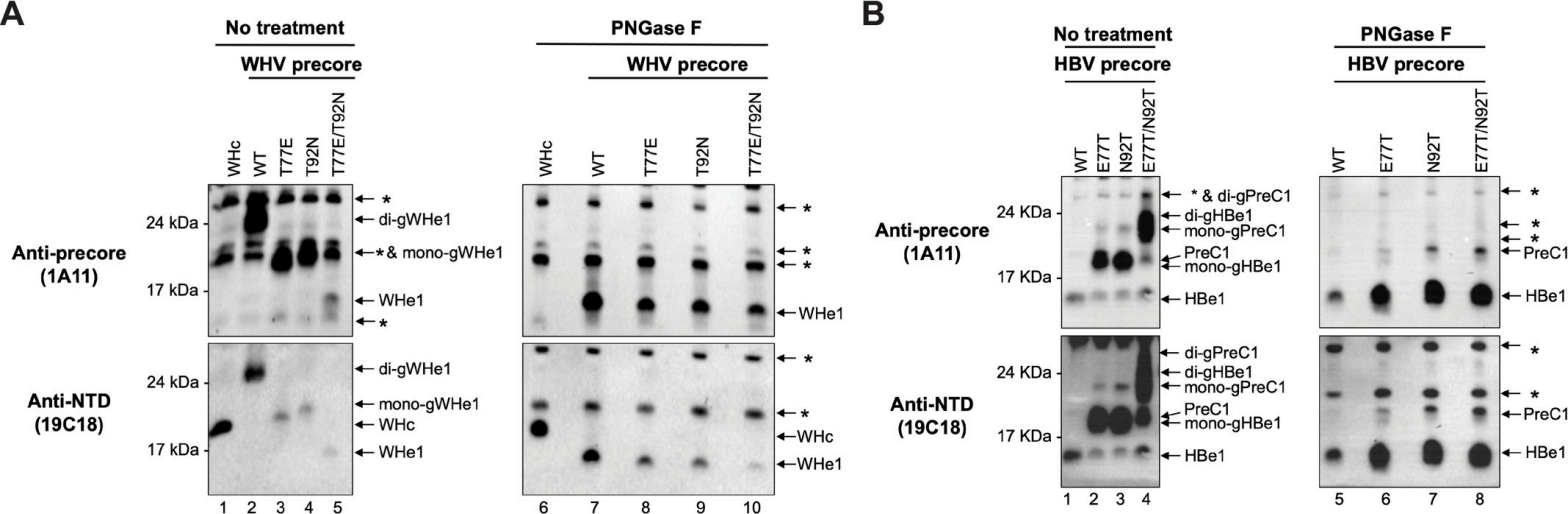
Precore N-glycosylation enhanced the secretion of precore gene products. Immunoblot analysis of **(A)** WHV and **(B)** HBV precore gene products in the culture supernatant of Huh7 cells transfected with WHV or HBV precore constructs. The supernatants were concentrated by ultrafiltration and treated with PNGase F or not and resolved by regular SDS-PAGE, followed by sequential immunoblotting with mAb 1A11 and 19C18 on the same membrane. WHV core-transfected cell culture supernatant served as the control for the background bands. WHe1, WHeAg that has the putative cleavage site on the 159^th^ residue at CTD (**Fig 1A**) as identified previously [28]; mono-gWHe1, mono-glycosylated WHe1; di-gWHe1, doubly-glycosylated WHe1; WHc, WHV core protein; HBe1, HBeAg that has the putative cleavage site on the 154^th^ residue at CTD (**Fig 1A**); mono-gHBe1, mono-glycosylated HBe1; di-gHBe1, doubly-glycosylated HBe1; PreC1, PreC antigen as previously characterized (**Fig 1A**); mono-gPreC1, mono-glycosylated PreC1; di-gPreC1, doubly-glycosylated PreC1. *, cross-reactive background bands.

In eukaryotic systems, the NXT/S sequon is necessary but not sufficient for N-glycosylation, and only approximately 60% of such sequons are glycosylated on secretory proteins [50]. Since there are two putative N-glycosylation motifs ^75^NIT^77^ and ^90^NDT^92^ in the WHV precore protein (**Fig 1A**), we firstly examined which of these two sites is N-glycosylated. For this purpose, we generated WHV precore expression constructs including the wild-type (WT) and three substitution mutants to mimic the corresponding sequences in the HBV precore gene, namely T77E, T92N, and T77E/T92N, which lose either one or both N-glycosylation sequons. We analyzed the secreted WHeAg from transfected human hepatoma Huh7 cells by SDS-PAGE and western blot analysis, using two mAbs, 1A11 and 19C18, targeting the unique -10 N-terminal extension or the shared NTD region, respectively (**Fig 1A**). We found that WHeAg expressed from both the T77E and T92N mutant constructs migrated faster than WT (**Fig 2A**, lanes 3, 4 vs. 2), and WHeAg expressed from the T77E/T92N double mutant construct migrated even faster (**Fig 2A**, lane 5 vs. 3, 4). These results suggested that both motifs were likely glycosylated. To further confirm that the slow mobilities of the WHeAg mutants were caused by N-glycosylation, we performed PNGase F treatment, which removes N-linked glycans from glycoproteins. After PNGase F treatment, we found that WT, T77E, T92N, and T77E/T92N WHeAg all migrated to the same positions as the untreated T77E/T92N WHeAg (**Fig 2A**, lanes 7–10), indicating that the two sequons, ^75^NIT^77^ and ^90^NDT^92^, were efficiently N-glycosylated during WHV precore processing and secretion. Interestingly, we found that the mutants losing either one of the sequons showed decreased WHeAg secretion compared to the WT (**Fig 2A** lane 2 vs. 3, 4; lane 7 vs. 8, 9), and the level of T77E/T92N WHeAg which lost both sequons was even lower than that of T77E or T92N WHeAg (**Fig 2A** lane 5 vs. 3, 4; lane 10 vs. 8, 9), indicating that N-glycosylation on the WHV precore gene enhanced WHeAg secretion and that the two sequons acted additively or synergistically. In addition, we demonstrated that N-glycosylation also enhanced the secretion of WHV precore gene products in the woodchuck hepatoma WC3 cells (**S2 Fig**).

Although no N-linked glycosylation site is present in the HBV precore/core genes, the presence of N75 and N90 in ^75^NLE^77^ and ^90^NTN^92^ on the HBV precore, which are located on the protein surface (**Fig 1C**), suggested that substitution of E77 and N92 with Thr might confer N-glycosylation to the HBV precore. To examine whether HBV precore could be glycosylated after introducing N-glycosylation motifs, we made substitutions of E77 and N92 in the HBV precore for the corresponding T77 and T92 in the WHV precore. We found that E77T and N92T HBeAg migrated slower on SDS-PAGE compared to the WT HBeAg (**Fig 2B**, lanes 2, 3 vs. 1), suggesting that E77T and N92T HBeAg was mono-glycosylated. Furthermore, E77T/N92T HBeAg migrated even slower than E77T and N92T HBeAg (**Fig 2B**, lane 4 vs. 2, 3), indicating that E77T/N92T HBeAg was di-glycosylated. In addition to HBeAg, we recently reported that the PreC antigen, which retains the N-terminal signal peptide, is another secretory product of the HBV precore gene [16]. Intriguingly, E77T or N92T PreC antigen also migrated slower than the non-glycosylated WT PreC and the E77T/N92T PreC migrated even slower (**Fig 2B**, lanes 2, 3 vs. 1), indicating that PreC antigen, like the HBeAg, could also be glycosylated after introducing the N-glycosylation motifs into the HBV precore gene. N-glycosylation was further confirmed by PNGase F treatment, which caused E77T, N92T, and E77T/N92T HBeAg or PreC antigen to migrate to the same position as the WT HBeAg or PreC antigen, respectively (**Fig 2B**, lanes 5–8). These results thus not only demonstrated that the secretory products of the HBV precore gene could be glycosylated after introducing N-glycosylation motifs but also indicated that the biogenesis of PreC antigen, like HBeAg, needs to go through the secretory pathway, which is consistent with our previous finding that N-glycosylated WHV PreC antigen was detected in the blood of WHV-infected woodchucks [28]. Moreover, we observed that all HBV precore mutants had increased HBeAg and PreC secretion compared to the WT precore (**Fig 2B**, lanes 6–8 vs. 5). Altogether, our data collectively demonstrated that N-glycosylation enhanced the levels of secreted precore gene products in the context of both HBV and WHV.

### Loss of WHV precore glycosylation sequons showed modest effects on WHc functions in capsid assembly and pgRNA packaging

**Fig 3:**
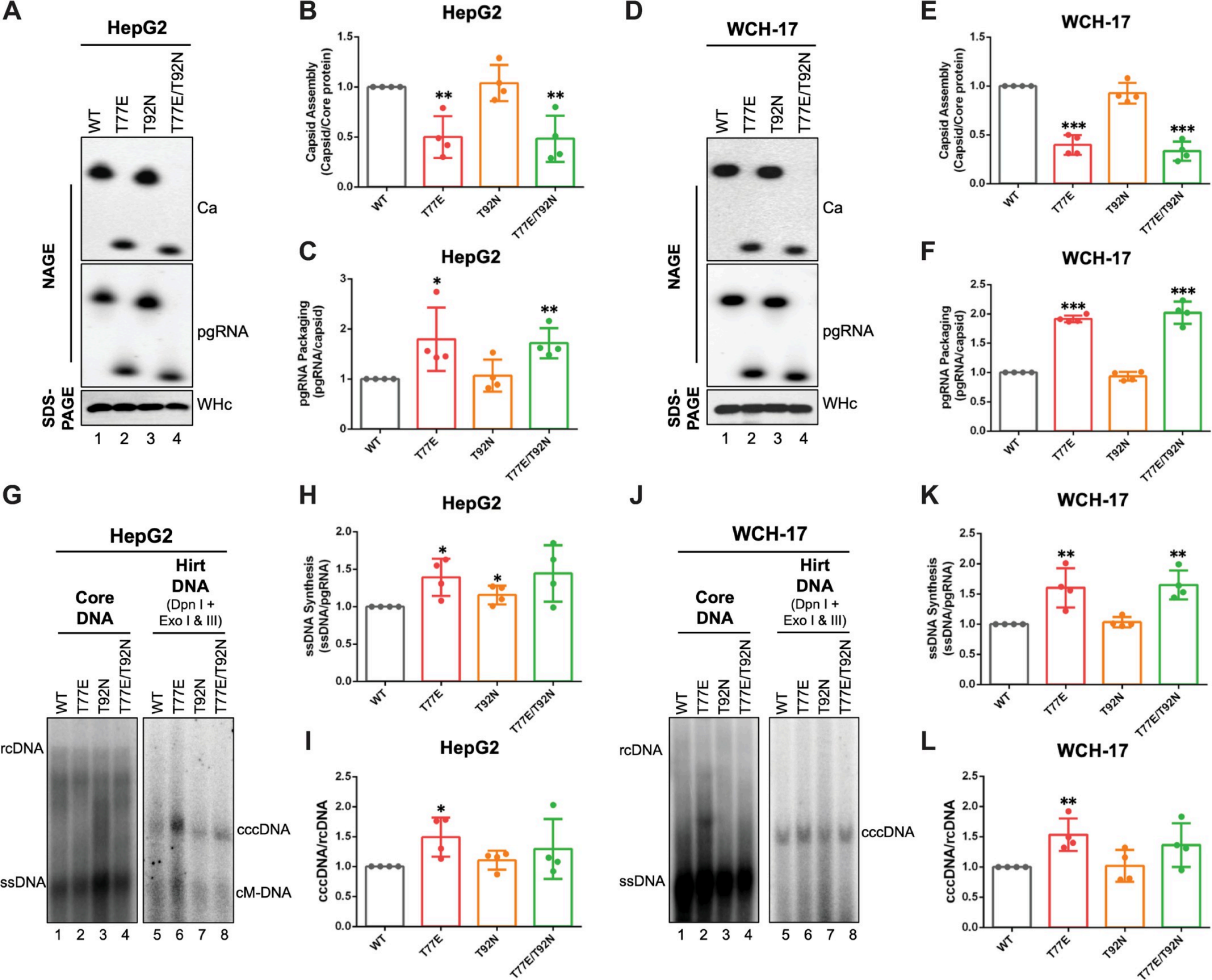
Effects of eliminating N-glycosylation sequons on WHc functions. The WHV replicon construct expressing the T77E, T92N, or T77E/T92N WHc mutants, or WT WHc was transfected into HpeG2 or WCH-17 cells. **(A)** The assembled capsids (top) and packaged pgRNA (middle) were detected by the C33 anti-HBc/WHc mAb and anti-sense WHV RNA probe, respectively, following the resolution of cytoplasmic lysates from the transfected HepG2 cells by native agarose gel electrophoresis (NAGE) and transfer to nitrocellulose membrane. Levels of WHc proteins (bottom) were measured by western blot using 19C18 anti-HBc/WHc mAb after SDS-PAGE. Capsid assembly efficiency **(B)** was determined by normalizing the levels of capsids to those of total WHc protein, and pgRNA packaging efficiency **(C)** was determined by normalizing the levels of pgRNA to those of capsids, with the efficiencies of WT WHc set to 1.0. **(D)-(F)** Cytoplasmic lysates from WHV replicon transfected WCH-17 cells were analyzed for capsid assembly and pgRNA packaging, as described for HepG2 cells. **(G)** WHV core DNA was released from the NCs of cytoplasmic lysate by SDS-proteinase K treatment and detected by Southern blot analysis. WHV PF-DNA was extracted from the transfected cells by the Hirt extraction method. The extracted DNA was treated with Dpn I plus the exonucleases I and III (Exo I & III) to remove all DNA with free 3’ ends. The ssDNA synthesis efficiency **(H)** was determined by normalizing the levels of ssDNA to those of pgRNA in (**A**), and the cccDNA formation efficiency **(I)** was determined by normalizing the levels of cccDNA to those of rcDNA in **(G)**, with the efficiencies of WT WHc set to 1.0. Similarly, core DNA and PF-DNA from WHV replicon transfected WCH-17 cells were analyzed **(J)-(L)**. Data is shown as mean ± SD. Two-tailed unpaired Student’s t test was used to compare the difference of each dataset versus WT WHc (*, *p* < 0.05; **, *p* <0.01; ***, *p* < 0.001). Ca, capsid; pgRNA, pregenomic RNA; WHc, WHV core protein; ssDNA, single-strand DNA; rcDNA, relaxed circular DNA; cccDNA, covalently closed circular DNA; cM-DNA, closed minus strand DNA.

Hepadnaviral precore protein is not necessary for viral replication [26,27,31]; however, any changes in the core region of the coding sequence for precore protein will also necessarily lead to the same changes in the core protein, which might affect core protein functions, e.g., in capsid assembly, pgRNA packaging, reverse transcription, cccDNA formation, or virion secretion. To test the effects of N-glycosylation motif loss on WHc function, we replaced T77 and T92 in the WHV precore/core with the E77 and N92 found in the HBV precore/core in a WHV construct (i.e., replicon) that supports WHV replication [37]. We first analyzed WHc expression, capsid assembly, and pgRNA packaging in replicon transfected human and woodchuck hepatoma cells. We found that none of these mutations affected WHc expression levels in HepG2 cells as determined by SDS-PAGE and western blot analysis (**Fig 3A**). On the other hand, T77E and T77E/T92N capsids migrated faster than WT and T92N capsids after native agarose gel electrophoresis (NAGE) (**Fig 3A**, lanes 2, 4 vs. 1, 3), which was expected as T77E introduces an additional negative charge on each of the 240 WHc subunits on the capsid surface (**Fig 1B**). When we normalized the capsid amount to the core protein amount as a measure of capsid assembly, we found that T77E and T77E/T92N mutants showed a ca. 2-fold decrease in capsid assembly compared to WT and T92N mutant (**Fig 3B**). Normalization of the levels of pgRNA packaging to the capsid levels showed that T77E and T77E/T92N mutants had a slight increase (1.5-fold) in pgRNA packaging efficiency than WT and T92N mutant (**Fig 3C**). Similarly, we obtained the same results in two woodchuck hepatoma cell lines, WCH-17 (**Fig 3D–3F**) and WC3 (**S3A–S3C Fig**), as well as in another human hepatoma cell line, Huh7 (**S3D–S3F Fig**). It is important to note that steady-state levels of viral capsids and packaged pgRNA, not assembly or pgRNA packaging *per se*, were measured here. It is formally possible that the changes in the state-levels of these parameters (and others measured below such as viral core DNA and cccDNA levels) observed here could be due to the modulation of disassembly/degradation/turnover of these parameters as well as (or even instead of) their assembly/production. For the sake of simplicity, we chose to describe the results here in terms of assembly/production and future studies may be warranted to directly examine the effects of assembly/production vs. disassembly/degradation/turnover of the various parameters.

### WHc mutants lacking the precore glycosylation sequons remained competent for reverse transcription and cccDNA amplification

To study the effects of the WHc mutations on WHV reverse transcription, we analyzed WHV DNA released from cytoplasmic NCs (i.e., core DNA) in transfected human and woodchuck hepatoma cells by Southern blot analysis. Similar to WT WHc, all WHc mutants were competent in core DNA synthesis (i.e., reverse transcription) (**Figs 3G**, **3J, S3G and S3I**), and T77E and T77E/T92N slightly increased ssDNA synthesis (**Figs 3H**, **3K, S3H and S3J**) upon normalization to pgRNA packaging levels. Interestingly, we found that the core DNA patterns above the ssDNA (mostly partially double-stranded DNA) of T77E and T92N were different from WT while the pattern of T77E/T92N was similar to WT(**Figs 3G and S3I**), suggesting that the individual T77E and T92N WHc mutations may have altered the kinetics of plus-strand DNA synthesis while the double T77E/T92N substitutions abrogated the effect of each other. Because the rcDNA and other partially double-stranded DNA replication intermediates (migrating above ssDNA) from the either the WT or mutant WHV replicons were barely detected in woodchuck hepatoma cells (**Figs 3J and S3G**), we were unable to discern any potential effects of the mutations on plus strand DNA synthesis in woodchuck cells, in contrast to human cells. To investigate the effects of the WHc mutations on cccDNA formation, we extracted extrachromosomal DNA (i.e., protein-free DNA or PF-DNA, including viral DNA without the attached RT protein as well as plasmids) from the WHV replicon-transfected human and woodchuck hepatoma cells by Hirt extraction and detected cccDNA after Dpn I digestion followed by exonuclease I and III (Exo I/III) treatment, which removes plasmid DNA as well as non-covalently closed viral and cellular DNA [37,51]. We found that the WHc mutations did not affect cccDNA levels via the intracellular amplification pathway although T77E showed a slight increase in cccDNA formation (**Figs 3G**, **3I**, **3J**, **3L, S3I and S3K**). We found that ssDNA was the predominant species of WHV DNA inside NCs from the replicon-transfected cells (**Figs 3 and S3**), and that the WHV rcDNA was barely detected in woodchuck hepatoma cells but was more clearly detected in human hepatoma cells (**Figs 3G**, **S3I** vs. **S3J and S3G**). However, we could not detect WHV virion secretion from the transfected cells, with either the WT or mutant WHc, by Southern blot analysis, even after CsCl gradient fractionation (**S4 Fig**), similar to what we have reported recently [37]. Altogether, our results indicated that WHc mutations lacking the N-glycosylation sequons remained competent in reverse transcription and cccDNA formation but any effects on virion secretion could not be assessed in the available cell culture systems.

### HBc mutants with precore glycosylation sequons showed no defects in capsid assembly, pgRNA packaging, or reverse transcription

**Fig 4:**
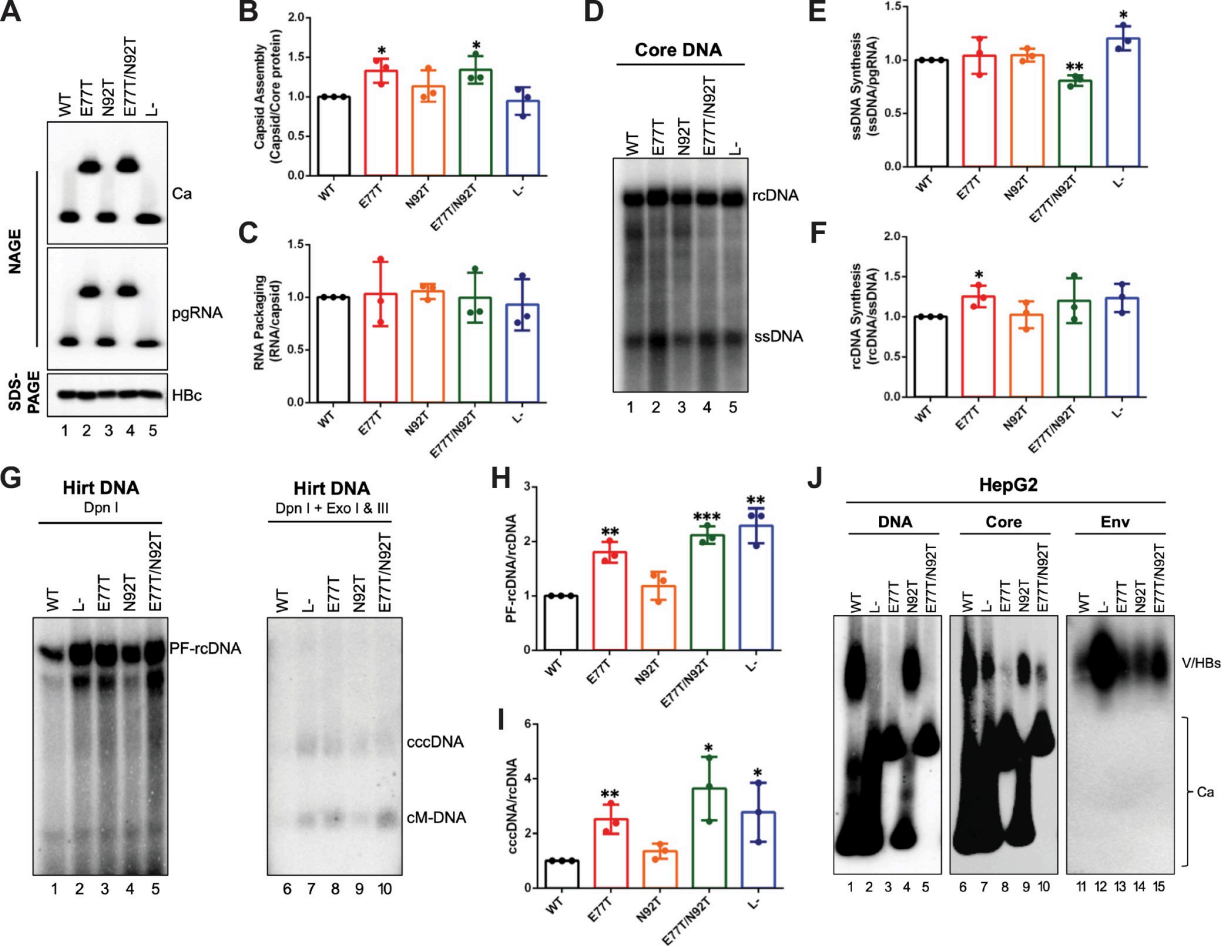
Effects of introducing N-glycosylation sequons on HBc functions. The HBV replicon construct expressing the E77T, N92T, or E77T/N92T mutant HBc, or WT HBc, or L^-^ that is defective in L-HBs expression was transfected into HepG2 cells. **(A)** The assembled capsids (top) and packaged pgRNA (middle) were detected by the T2221 anti-HBV precore/core NTD mAb and anti-sense HBV RNA probe, respectively, following the resolution of cytoplasmic lysates by NAGE and transfer to nitrocellulose membrane. Levels of HBc proteins (bottom) were measured by western blot using T2221 mAb after SDS-PAGE. Quantitative results of capsid assembly efficiency **(B)** and pgRNA packaging efficiency **(C)** were obtained as described in **Fig 3** and are shown. **(D)** HBV core DNA was released from the NCs of cytoplasmic lysate by SDS-proteinase K treatment and detected by Southern blot analysis. The ssDNA synthesis efficiency **(E)** was determined by normalizing the levels of ssDNA to those of pgRNA in (**A**), and the rcDNA synthesis efficiency **(F)** was determined by normalizing the levels of rcDNA to those of ssDNA in **(D)**, with the efficiencies of WT HBc set to 1.0. **(G)** HBV PF-DNA was extracted from the transfected cells by the Hirt extraction method. The extracted DNA was treated with Dpn I (left) or Dpn I plus Exo I & III (right) to remove all DNA with free 3’ ends. The PF-rcDNA synthesis efficiency **(H)** was determined by normalizing the levels of PF-rcDNA to those of rcDNA in **(D)**, and cccDNA formation efficiency **(I)** was determined by normalizing the levels of cccDNA to those of rcDNA in **(D)**, with the efficiencies of WT HBc set to 1.0. **(J)** Culture supernatant from HBV replicon transfected HepG2 cells was harvested at day 5 post transfection, concentrated (50X), resolved by agarose gel electrophoresis and transferred to nitrocellulose membrane. HBV DNA, capsids in virions, and envelope proteins in virions and subviral particles were detected sequentially using a ^32^P-labeled HBV DNA probe, anti-HBV precore/core NTD mAb T2221, and anti-HBs polyclonal antibody, respectively, on the same membrane. Data is shown as mean ± SD. Two-tailed unpaired Student’s t test was used to compare the difference of each dataset versus WT HBc (*, *p* < 0.05; **, *p* <0.01; ***, *p* < 0.001). Ca, capsid; pgRNA, pregenomic RNA; HBc, HBV core protein; ssDNA, single-strand DNA; rcDNA, relaxed circular DNA; cccDNA, covalently closed circular DNA; cM-DNA, closed minus strand DNA; Ca, capsid; V, virion; HBs, HBV surface antigen.

We asked next whether the introduction of N-glycosylation sequons would affect HBc functions. We substituted the residues on the HBV precore/core protein (E77 and N92) with the corresponding WHV residues (T77 and T92) and transfected the HBV WT and mutant replicons into HepG2 cells. We also included the L^-^ mutant that is defective in L envelope protein (L-HBs) expression as the negative control for complete virion (i.e., DNA-containing virion) secretion (see below). Our results showed that the HBc expression levels were not affected by the introduction of either or both glycosylation sites (**Fig 4A**). Interestingly, we found that the HBc E77T and E77T/N92T mutants slightly increased capsid levels compared to WT and N92T mutant (**Fig 4A and 4B**), an opposite result of the reverse T77E and T77E/T92N mutations in WHc (**Figs 3 and S3**), further indicating a role of E77 in enhancing capsid assembly. Contrary to WHc, we found that none of the HBc mutations affected pgRNA packaging (**Fig 4C**). Noticeably, the capsids of E77T and E77T/N92T migrated slower than WT and N92T capsids in the NAGE assay (**Fig 4A**, lanes 2, 4 vs. 1, 3), which was opposite to the capsids of WHc T77E and T77E/T92N that migrated faster than WT and T92N capsids (**Figs 3 and S3**), and which could be explained by the loss of negative charges on the capsid surface of these mutants. We also found that none of the mutants showed any major defects in reverse transcription, including ssDNA and rcDNA synthesis (**Fig 4D–4F**). Thus, our results indicated that all HBc mutants remained competent for capsid assembly, pgRNA packaging, and DNA synthesis.

### HBc E77T increased intracellular cccDNA amplification and decreased virion secretion

Next, we examined the effects of the HBc mutations on cccDNA formation in HepG2 cells. Interestingly, we found that E77T and E77T/N92T showed increased PF-rcDNA and cccDNA levels compared to WT (ca. 2- to 3-fold), which was similar to the L^-^ mutant that is defective in DNA-containing virion (i.e., complete virion) secretion (**Fig 4G–4I**). On the other hand, N92T showed comparable levels of PF-rcDNA and cccDNA as the WT (**Fig 4G–4I**). We then asked whether these HBc mutations could affect capsid-envelope interactions during virion secretion. We analyzed virion secretion in the cell culture supernatant by NAGE. We found that E77T and E77T/N92T mutants showed a dramatic decrease in DNA-containing virions (**Fig 4J**, lanes 3, 5), whereas N92T secreted complete virions at a level similar to WT (**Fig 4J**, lane 4). In contrast to the inability to support complete virion secretion, the L^-^ mutant still could support the secretion of empty virions as detected by the virion-associated capsid [13], albeit at reduced levels compared to the WT (**Fig 4J**, lane 2, 7), as we reported before [14]. The E77T and E77T/N92T mutants also showed severe defects in the secretion of empty virions (**Fig 4J**, lanes 8, 10). Thus, the decreased secretion of complete and empty virions by the E77T and E77T/N92T mutants suggested that HBc E77 is critical for HBV capsid-envelope interaction and/or virion secretion. We obtained the same results in another human hepatoma cell line Huh7 (**S5A Fig**). When we prepared viral inoculum using a large-scale transfection of Huh7 cells, we could detect low-level secretion of complete virions by E77T and E77T/N92T that were much lower than those of WT and N92T, suggesting that E77T impaired but did not completely block virion secretion (**S5B Fig**). The increased cccDNA amplification by E77T and E77T/N92T described above (**Fig 4G**) was thus most likely due to impaired virion secretion, similar to the known effect of the L^-^ mutant on cccDNA amplification.

### Both HBc E77T and N92T mutants impaired cccDNA formation during *de novo* infection

**Fig 5:**
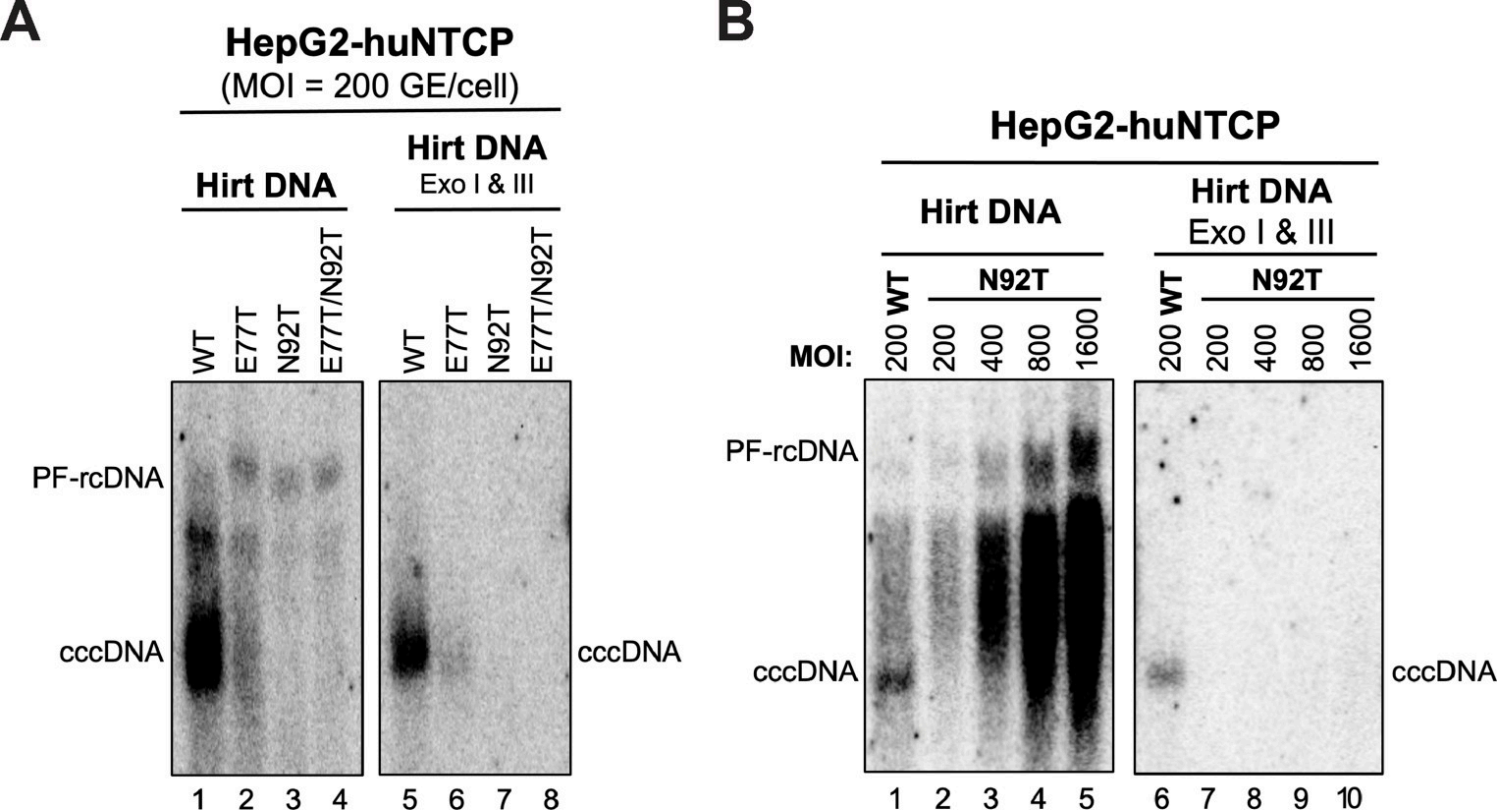
Effects of introducing N-glycosylation sequons on *de novo* HBV infection. HBV inocula were prepared from HBV replicon transfected Huh7 cells. **(A)** Infection of WT, E77T, N92T, and E77T/N92T mutant virus (MOI = 200 GE/cell) in HepG2-huNTCP cells. PF-DNA was extracted from infected cells at 4 dpi by the Hirt extraction method and analyzed by Southern blot analysis, without (lanes 1–4) or with pretreatment by Exo I & III (lanes 5–8). **(B)** Infection of N92T mutant virus with increased dosage (MOI = 200, 400, 800, or 1600 GE/cell) in HepG2-huNTCP cells. PF-DNA was extracted from infected cells at 4 dpi and analyzed by Southern blot analysis as described above. PF-rcDNA, protein-free rcDNA; cccDNA, covalently closed circular DNA.

To investigate the infectivity of HBc mutant viruses, we infected HepG2-huNTCP cells with inocula prepared from Huh7 cells transfected with HBc WT, E77T, N92T, and E77T/N92T replicons. Because of the impaired secretion of E77T and E77T/N92T mutant virus, we increased the volume of these inocula to match the WT and N92T inocula in regard to the total amount of complete virions used (MOI = 200 GE/cell in all cases). After 4 days post-infection (dpi), we harvested the cells and extracted PF-DNA (including cccDNA) for Southern blot analysis. We also treated PF-DNA with Exo I and III to remove the PF-rcDNA and other viral DNAs that could interfere with the cccDNA detection. We found that E77T could support HBV infection, as indicated by cccDNA formation, but the cccDNA levels of E77T were much lower than those of the WT, indicating that the mutant was much less infectious than WT (**Fig 5A**, lanes 1, 5 vs. 2, 6). Moreover, N92T failed to form any detectable cccDNA during infection (**Fig 5A,** lanes 3, 7), and E77T/N92T was like N92T (**Fig 5A**, lanes 4, 8). To further confirm that N92T was defective in infection, we increased the N92T inoculum up to an MOI of 1600 GE/cell, which still failed to support any detectable cccDNA formation at 4 dpi and 9 dpi (**Figs 5B and S5C**). It is also noticeable that an intense PF DNA smear migrating between the PF-rcDNA and cccDNA accumulated with increasing amounts of the N92T inoculum (**Fig 5B**). As we reported recently [37], these heterogeneous PF-DNA species were likely degradation/processing products derived from rcDNA during the entry of the N92T mutant virus. As N92T showed no defect in cccDNA formation via the intracellular amplification pathway (**Fig 4G**) but was unable to support cccDNA formation during infection, it most likely had a block(s) before nuclear import of rcDNA-containing NCs, the common step shared for cccDNA formation between *de novo* infection and intracellular amplification.

### WHc supported more efficiently pgRNA packaging of WHV and cccDNA amplification of both HBV and WHV than HBc

**Fig 6:**
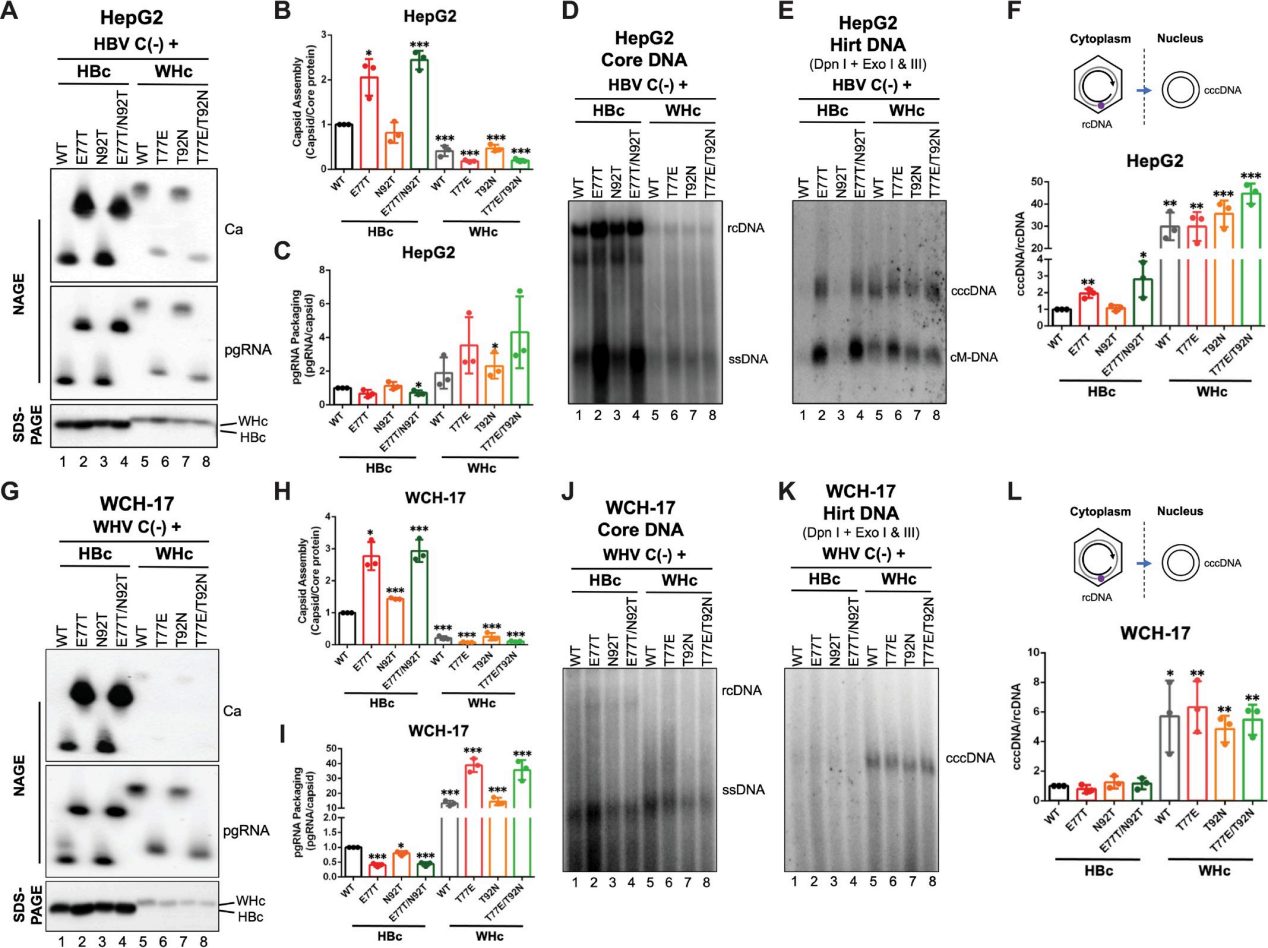
Effects of WT and mutant HBc or WHc on supporting the replication of HBV and WHV. The HBV replicon construct that is defective in HBc expression was co-transfected with WT and mutant HBc or WHc expression constructs in HepG2 cells. **(A)** The assembled capsids (top) and packaged pgRNA (middle) were detected by the C33 anti-HBc/WHc mAb and anti-sense HBV RNA probe, respectively, following the resolution of cytoplasmic lysates by NAGE and transfer to nitrocellulose membrane. Levels of HBc or WHc proteins (bottom) were measured by western blot using 19C18 anti-HBc/WHc mAb after SDS-PAGE. Quantitative results of capsid assembly efficiency **(B)** and pgRNA packaging efficiency **(C)** were obtained as described in **Fig 4** and are shown. **(D)** core DNA was released from the NCs of cytoplasmic lysate by SDS-proteinase K treatment and detected by Southern blot analysis. **(E)** PF-DNA was extracted by the Hirt extraction method. The extracted DNA was treated with DpnI plus Exo I & III to remove all DNA with free 3’ ends. The cccDNA formation efficiency **(F)** was determined by normalizing the levels of cccDNA to those of rcDNA, with the efficiency from WT HBc set to 1.0. Similarly, a WHV replicon construct that is defective in WHc expression was co-transfected with WT and mutant HBc or WHc expression constructs in WCH-17 cells. **(G)** The assembled capsids (top) and packaged pgRNA (middle) were detected by the C33 anti-HBc/WHc mAb and anti-sense WHV RNA probe, respectively. Levels of HBc or WHc proteins (bottom) were measured by western blot using 19C18 anti-HBc/WHc mAb after SDS-PAGE. Capsid assembly efficiency **(H)** and pgRNA packaging efficiency **(I)** were determined as described above and are shown. **(J)** core DNA and **(K)** PF-DNA from the transfected cells was analyzed by Southern blot analysis. **(L)** cccDNA formation efficiency of WHV was determined by normalizing the levels of cccDNA to those of rcDNA, with the efficiency from WT HBc set to 1.0. Data is shown as mean ± SD. Two-tailed unpaired Student’s t test was used to compare the difference of each dataset versus WT HBc (*, *p* < 0.05; **, *p* <0.01; ***, *p* < 0.001). Ca, capsid; pgRNA, pregenomic RNA; HBc, HBV core protein; WHc, WHV core protein; ssDNA, single-strand DNA; rcDNA, relaxed circular DNA; cccDNA, covalently closed circular DNA; cM-DNA, closed minus strand DNA.

WHc or HBc can support pgRNA packaging and reverse transcription of the heterologous virus [33–36]; however, the effects on cccDNA formation and virion secretion in such *trans*-complementation assays have not been comprehensively studied. Therefore, we firstly co-transfected the HBc or WHc expression construct together with an HBV replicon that is defective in HBc expression [HBV-C(-)] into human hepatoma HepG2 cells or woodchuck hepatoma WCH-17 cells. WHc-WT showed similar (in WCH-17 cells) or somewhat lower (in HepG2 cells) levels of capsid assembly than HBc-WT after normalization to the total core protein levels, although the WHc expression levels were always lower than those of HBc in both cell lines tested (**Figs 6A, 6B and S6**). In line with our full-length replicon transfection results (**Figs 3**, **4 and S3**), HBc-E77T and HBc-E77T/N92T increased capsid assembly compared with WT HBc, while WHc-T77E and WHc-T77E/T92N decreased capsid assembly compared with WT WHc in the *trans*-complementation assay in both cell lines (**Figs 6A**, **6B and S6**). WHc-WT and HBc-WT also supported similar levels of HBV pgRNA packaging in HepG2 cells (**Fig 6A and 6C**) but WHc was less effective than HBc in the woodchuck WCH-17 cells (**S6 Fig**), suggesting that species-specific host factors as well as the viral pgRNA and/or RT protein determine the efficiency of pgRNA packaging by HBc vs. WHc.

Both HBc and WHc supported HBV reverse transcription (**Fig 6D**). After normalization to the pgRNA packaging levels, WHc supported approximately the same or even higher levels of ssDNA synthesis than HBc (**Figs 6D and S6**). On the other hand, after normalizing to the ssDNA levels, the rcDNA levels supported by WHc were lower (by 3-10-fold) than by HBc in either the human or woodchuck cell lines. As it will be detailed below, this reduction of rcDNA was likely not due to a defect in plus strand elongation but rather due to the instability of mature NCs composed of WHc when compared with HBc. The gain or loss of the glycosylation sequons in the HBc or WHc, respectively, did not have strong effects on HBV pgRNA packaging or reverse transcription in the *trans*-complementation assay as noted above in the single replicon plasmid assay (**Fig 4**). Interestingly, after normalization to rcDNA levels, the direct comparison of WHc vs. HBc in the *trans*-complementation assay revealed ca. 30-fold higher levels of HBV cccDNA when supported by WHc-WT instead by HBc-WT in either HepG2 or WCH-17 cells (**Figs 6E**, **6F and S6**), suggesting that WHc increased HBV cccDNA formation regardless of host species. In addition, the HBc or WHc mutants behaved similarly to the corresponding WT HBc or WHc in supporting cccDNA amplification, except that the virion secretion defect of HBc E77T and E77T/N92T led to modestly enhanced cccDNA amplification (2–3 fold higher than WT), as expected in HepG2 cells (**Figs 6E**, **6F and S6G**). Because HBV virion secretion in the woodchuck cells was less efficient than that in the human cells [37], the effect of the HBc mutations on HBV cccDNA formation in the woodchuck WCH-17 cells was more subtle (less than 1.5-fold) (**S6 Fig**).

Given the similarities and differences observed above using either HBc or WHc to support HBV replication, we were then interested in determining how HBc could support WHV replication in comparison with WHc. We thus co-transfected the HBc or WHc expression construct together with the WHV replicon [WHV-C(-)] that is defective in WHc expression in WCH-17 and HepG2 cells. We found that HBc could support pgRNA packaging, reverse transcription, and cccDNA formation of WHV (**Figs 6G–6L** and **S7**). Interestingly, we observed a dramatic increase (by 5- to 35-fold) in WHV pgRNA packaging by WHc-WT and mutants compared to HBc-WT or mutants when complementing the WHV genome in both cell lines (**Figs 6I, S7C and S7G**). This was in contrast to the complementation of the HBV genome when WHc was either similar or less effective in supporting HBV pgRNA packaging as described above, further highlighting virus-specific interactions between the core protein and pgRNA and/or RT for controlling the efficiency of pgRNA packaging. On the other hand, we found that WHc supported 6-fold and 2-fold more WHV cccDNA formation in WCH-17 cells and HepG2 cells, respectively (**Figs 6L, S7L and S7G**), indicating that WHc supported more efficient cccDNA formation than HBc in the context of either HBV or WHV genome.

### Capsids formed by WHc failed to protect the rcDNA content

**Fig 7:**
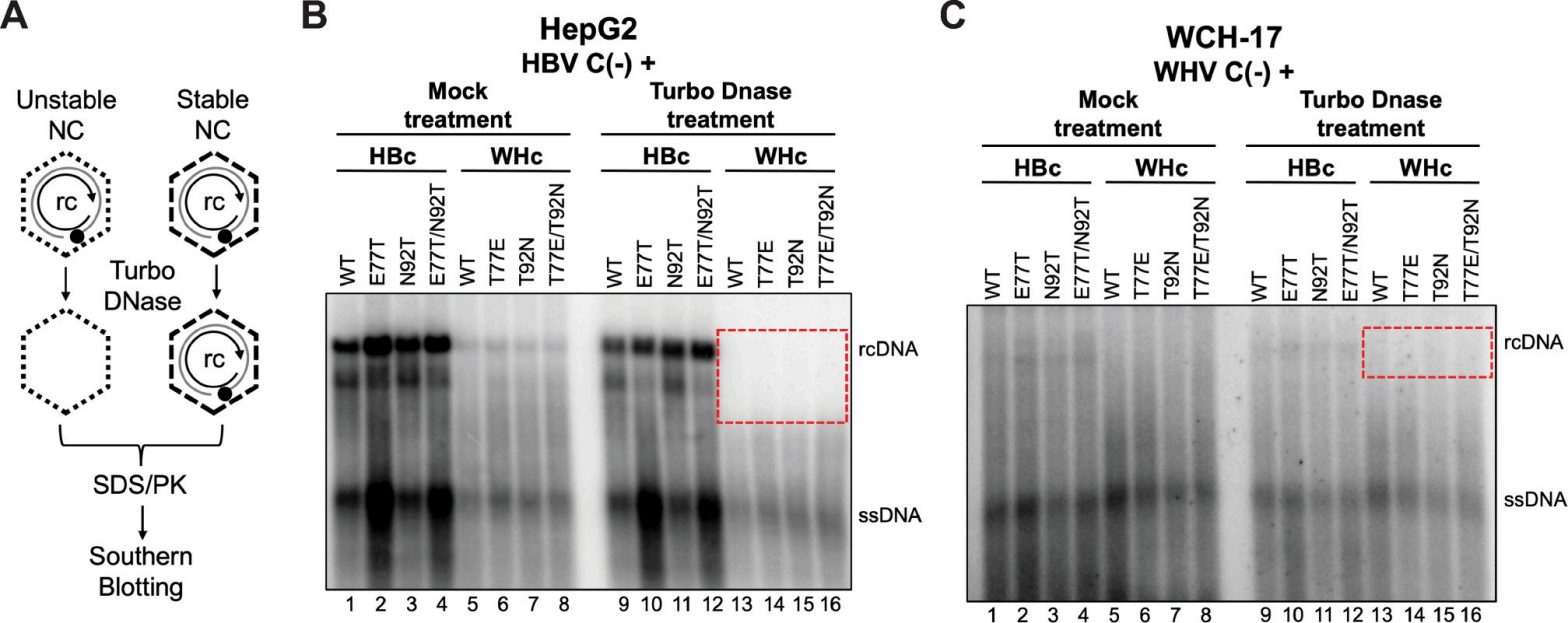
Effects of HBc or WHc on protecting the rcDNA content inside NCs. **(A)** schematic diagram of nuclease treatment of HBV or WHV NCs. Turbo DNase was used to degrade viral DNA inside unstable NCs. Following inactivation of the nuclease, NC-protected DNA was isolated and detected by Southern blot analysis. Core DNA released from the cytoplasmic lysate of WT and mutant HBc or WHc co-transfected with HBV-C(-) from HepG2 cells **(B)** or with WHV-C(-) from WCH-17 cells **(C)**, with or without prior Turbo DNase digestion, was detected by Southern blot analysis. The rcDNA signals obtained after Turbo DNase treatment is indicated by the dashed, red box for comparison to rcDNA signals present without nuclease treatment. ssDNA, single strand DNA; rcDNA, relaxed circular DNA.

Our recent studies indicated that the decreased stability of mature NCs can contribute to increased cccDNA amplification [52]. To test if the increased cccDNA supported by WHc vs. HBc was related to mature NC stability, we treated cytoplasmic lysate containing NCs with Turbo DNase, which degrades plasmid DNA as well as viral DNA if NCs are unable to protect their DNA content (**Fig 7A**) [52,53]. We found that Turbo DNase digestion removed HBV rcDNA, as well as DNA species migrating just below rcDNA (i.e., presumably partially double stranded DNA with plus strands close to completion but shorter than those present in rcDNA) from NCs formed by WHc (**Figs 7B and S8A**), suggesting that the NCs formed by WHc failed to protect rcDNA inside the mature (and near mature) NCs. Interestingly, we found that NCs formed by WT or mutant HBc did not show any major difference with or without Turbo DNase treatment (**Fig 7B**). Although only low levels of WHV rcDNA were detectable in the transfected cells, NCs formed by WT or mutant WHc but not by WT or mutant HBc also failed to protect the WHV rcDNA (**Figs 7C and S8B**), further suggesting that WHc-containing mature NCs were less stable than those containing HBc and regardless of whether the rcDNA was HBV or WHV. As we reported before [52], ssDNA in immature NCs was not affected significantly by Turbo DNase treatment (**Figs 7 and S8**).

### WHc proteins with or without precore glycosylation sequons failed to interact with HBV envelope proteins for virion secretion

**Fig 8:**
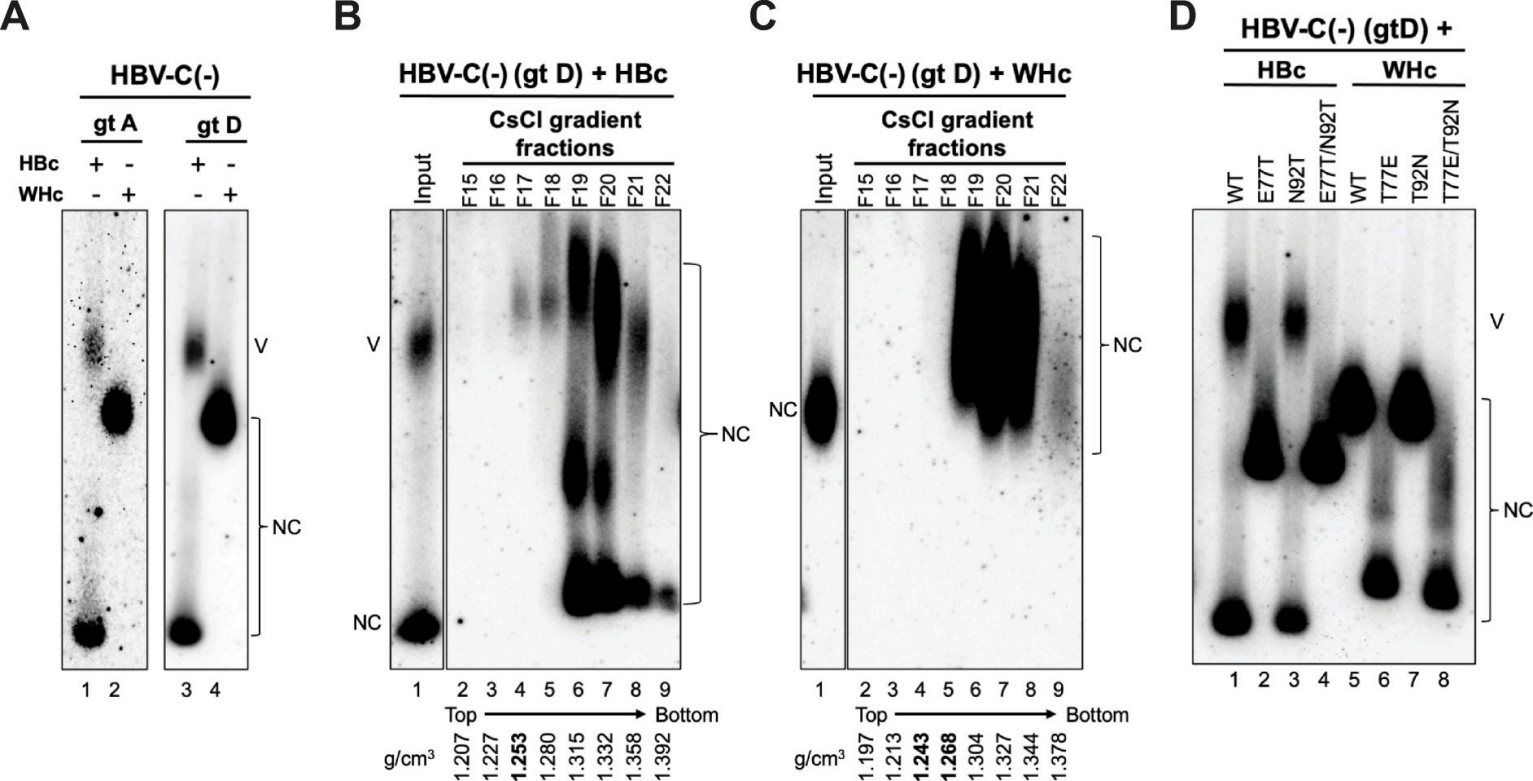
Effects of HBc or WHc on supporting HBV virion secretion. **(A)** concentrated culture supernatant of HepG2 cells co-transfected with WT HBc or WHc and HBV-C(-) (genotype A or D) was analyzed by agarose gel electrophoresis. HBV DNA in virions or naked capsids was detected by a HBV DNA probe. Concentrated culture supernatant of HepG2 cells co-transfected with WT HBc or WHc along with HBV-C(-) (genotype D) was fractionated by CsCl density gradient ultracentrifugation. The fractions from WT HBc **(B)** or WT WHc **(C)** co-transfected culture supernatant was analyzed by agarose gel electrophoresis. HBV DNA in virions or naked capsids was detected by a HBV DNA probe. The density profile of each fraction is indicated at the bottom. The density of HBV virions (1.250 g/cm^3^) is highlighted in bold. **(D)** concentrated cell culture supernatant of HepG2 cells co-transfected with HBV-C(-) (genotype D) and WT or mutant HBc or WHc was analyzed by NAGE and DNA in virions or naked capsids was detected by a HBV DNA probe. NC, nucleocapsid; V, virion.

Since WHc could support HBV intracellular replication, we asked whether WT or mutant WHc could support HBV virion secretion. We firstly tested whether WHc could support virion secretion after complementation with the HBV genome defective in HBc expression. In contrast to WT HBc, WT WHc did not support HBV virion secretion (**Fig 8A**), suggesting that capsids formed by WHc were unable to interact with HBV envelope proteins for virion secretion. We confirmed the inability of WHc to interact with HBV envelope proteins and to support HBV virion secretion by using a genotype A replicon (instead of the genotype D replicon that was used in all other experiments here) that is defective of HBc expression (**Fig 8A**). To detect virion secretion more conclusively, we employed CsCl density gradient fractionation to concentrate DNA-containing virions and to separate virions from naked NCs in the culture supernatant of WHc and HBV-C(-) co-transfected HepG2 cells. In contrast to HBc complemented with HBV-C(-) (**Fig 8B**), we still could not detect HBV virions with WHc capsids after CsCl gradient fractionation (**Fig 8C**). Since HBc E77T was defective in supporting virion secretion, we asked whether WHc mutations with either the T77E or T92N or with the T77E/T92N substitution could rescue the interaction of WHc with HBV envelope proteins to support virion secretion. We found that none of the WHc mutations could support HBV virion secretion (**Fig 8D**), suggesting that the virus-specific interactions between the HBV capsid and envelope proteins required for virion secretion could not be rescued by these substitutions in WHc to mimic HBc.

## Discussion

**Fig 9:**
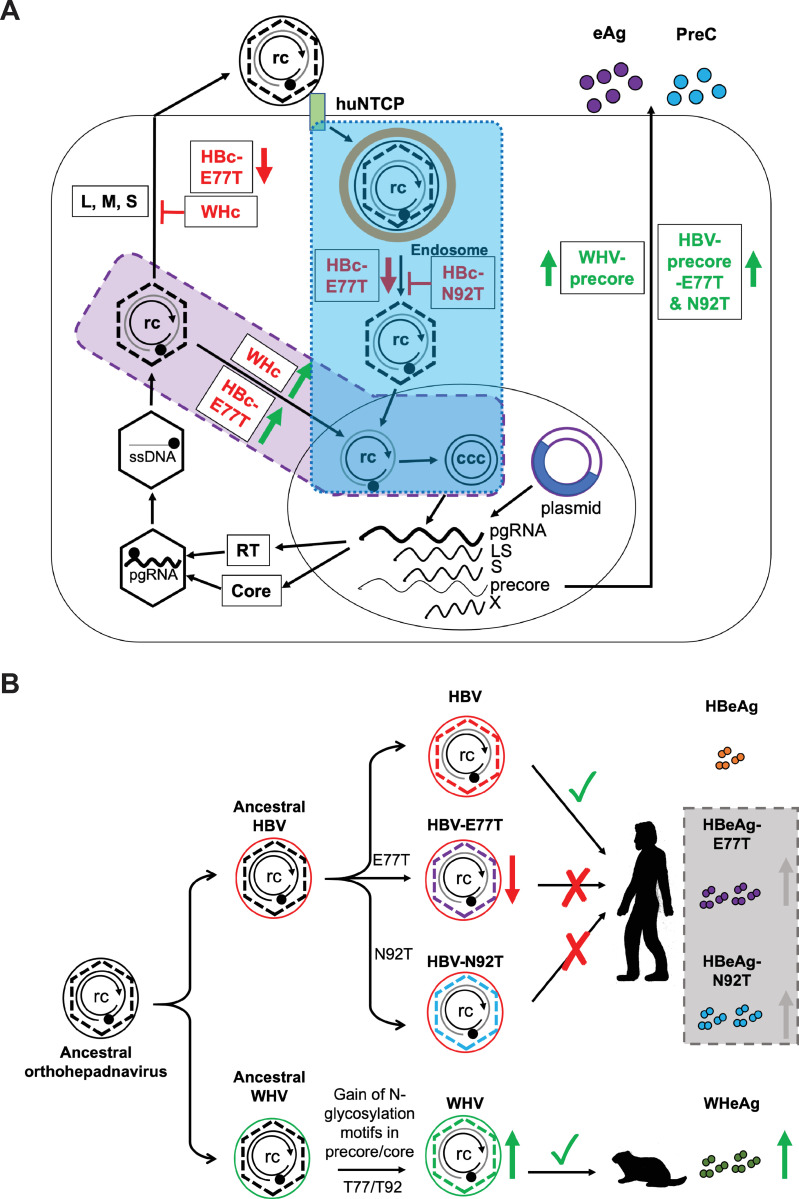
Summary of the roles of N-glycosylation motifs in precore/core genes on HBV or WHV precore and core functions and their implication in hepadnaviral evolution. **(A)** The N-glycosylation on the WHV or HBV precore proteins increased the secretion of e and PreC antigens (eAg and PreC). The E77T mutant that introduced the N-glycosylation sequon in HBc decreased HBV virion secretion and cccDNA formation (i.e., infectivity) during infection (blue box), while HBc-E77T increased cccDNA formation via the intracellular amplification pathway (purple box). The N92T mutant that introduced another N-glycosylation sequon on HBc showed no cccDNA formation during infection (blue box). When a HBV replicon that is defective in HBc expression was complemented with WT or mutant WHc, no HBV virion secretion was detected. However, WHc increased cccDNA formation via the intracellular amplification pathway (purple box) due to the decreased stability of mature NCs. **(B)** Proposed model of acquisition of N-glycosylation motifs in the WHV precore/core genes but not in HBV precore/core genes during the evolution. See text for details.

In this study, we investigated the roles of the N-glycosylation sequons in the overlapping hepadnaviral precore/core genes on the functions of precore and core proteins from both WHV and HBV. We found that N-glycosylation could enhance the levels of secreted precore gene products in the context of both WHV and HBV (**Fig 9A**). In terms of WHc or HBc functions, we found that the WHc-T77E substitution decreased capsid assembly while the HBc-E77T substitution increased capsid assembly. On the other hand, the HBc-E77T substitution was defective in supporting virion secretion, whereas HBc-E77T and HBc-N92T substitutions severely reduced and abolished cccDNA formation, respectively, during infection (**Fig 9A**). *Trans*-complementation experiments showed that both HBc and WHc could support all intracellular replication steps of the heterologous virus; however, WHc supported WHV pgRNA packaging more efficiently than HBc. Strikingly, WHc also supported much more efficiently cccDNA formation than HBc in the context of either HBV or WHV, which correlated with a decreased stability of mature nucleocapsids formed by WHc (**Fig 9A**). On the other hand, WT WHc failed to interact with HBV envelope proteins for virion secretion, and neither WHc-T77E nor WHc-T92N or WHc-T77E/T92N could rescue this defect (**Fig 9A**).

N-glycosylation, which is critical for proper protein folding in the secretory pathway, is generally associated with increased protein secretion [30,54,55]. Consistent with our previous observation that WHeAg in chronically WHV-infected woodchuck serum is an order of magnitude higher than the corresponding HBeAg in chronically HBV-infected patient serum [28], our mutagenesis analysis on the WHV and HBV precore genes further confirmed that N-glycosylation indeed increased the levels of secreted precore gene products. Intriguingly, seroconversion of WHeAg rarely occurs during chronic WHV infection in woodchucks [5], implicating a functional importance of N-glycosylation on WHeAg *in vivo* possibly by enhancing the WHeAg and PreC levels in the blood. It is also possible that N-linked glycans on WHeAg shield immunogenic epitopes [30]. The exact functions of WHV precore N-glycosylation, especially in facilitating persistent infection, warrants further investigation in woodchucks.

Although WHV is the first hepadnavirus that was discovered after the identification of HBV [4], *in vitro* studies of WHV remain limited [56–58]. In this study, we found that the WHc-T77E mutation decreased modestly (by ca. 50%) the levels of assembled capsids but not those of the packaged pgRNA, indicating that this mutation reduced the levels of empty capsids (i.e., without the viral pgRNA) (see also below). Although WHV rcDNA was barely detectable in cell culture, consistent with a previous report [37], we could readily detect WHV cccDNA following WHV replicon transfection of human and woodchuck hepatoma cells (i.e., intracellular cccDNA amplification). Whether WHV replicon-transfected cells can support virion secretion remains controversial [38,56,59]. Here, we were unable to detect WHV virions from the concentrated cell culture supernatant by Southern blot analysis, even with prior CsCl gradient fractionation, which is consistent with previous studies [37,38]. Due to this limitation, we were unable to study the effects of WHc mutations on WHV virion morphogenesis. On the other hand, injections of the cell culture supernatant from WHV replicon transfected HepG2, NIH3T3, and turkey embryonic fibroblasts caused productive infections in woodchucks [56,60], suggesting that WHV virions are secreted from cell cultures at low levels but were below the detection limit of our assays. The lack of WHV PreS1 promoter activity in liver-derived cell lines [38], possibly due to the loss of cell differentiation, could lead to a deficiency in L WHV envelope protein expression in these cells, which would explain the inefficient WHV virion secretion. Furthermore, the lack of an efficient *in vitro* system for WHV infection hampered this study on the infectivity of the WHV precore/core mutants. Our attempts to infect primary woodchuck hepatocytes *in vitro* [61] have not been successful for reasons yet to be elucidated.

In contrast, the well-established cell culture systems for HBV infection allowed us to study in detail the effects on HBc functions during the entire HBV lifecycle after introduction of the N-glycosylation sequons. Obviously, the core protein cannot be N-glycosylated even after the introduction of the N-glycosylation sequons into its sequence because it is made in the cytosol. We found that HBc-E77T increased capsid assembly, which was opposite to WHc-T77E, further suggesting that T77 is critical for capsid assembly. As the majority of HBV capsids (ca. 90%) formed during replication *in vitro* or *in vivo* are empty, the effect of T77 on capsid assembly was likely directed towards empty capsids. Indeed, introduction of one or both N-glycosylation sequons into HBc had little effect on the absolute levels of pgRNA that were packaged into HBV nucleocapsids (i.e., without normalizing to capsid levels), similar to the lack of an effect on pgRNA packaging into WHV nucleocapsids by the loss of these sequons. Interestingly, we found that HBc-E77T decreased both complete and empty HBV virion secretions. E77 is located on the tip of HBV capsid spike (**Fig 1B**). A previous cryo-EM analysis suggested that E77 is the residue that is involved in the interaction of the nucleocapsid with the viral envelope via charge interaction [62], which is consistent with our results. Another mutagenesis study indicated that the HBc-E77A mutation does not affect virion secretion [32]; however, the virion secretion assay used in that study may not been quantitative enough. Thus, it is possible that HBc-E77A also decreases virion secretion to some extent. On the other hand, HBc-N92T behaved similarly to the HBc-WT, in terms of post-entry steps of replication including cccDNA formation and virion secretion in transfected HepG2 cells, similar to the lack of an effect by the reverse T92N substitution in WHc on intracellular replication steps of WHV.

Interestingly, we found that HBV carrying the HBc-E77T substitution showed much reduced cccDNA levels and HBc-N92T showed no detectable cccDNA during *de novo* infection, indicating that both mutations affect one or more steps during the infection that are required for cccDNA formation by HBV. Consistent with our previous studies on HBc mutations in its NTD and the linker [63–65], our results here clearly demonstrated again that HBc mutations can affect cccDNA formation differentially via intracellular amplification vs. infection. HBc-D78S, the mutation at the capsid spike tip next to E77, was shown to cause dimer destabilization and structural deformation allosterically [66]. In analogy, the E77T substitution here might cause a premature destabilization of nucleocapsids during infection and thus impair virion infectivity but future studies will be required to elucidate how E77T exactly reduces infectivity. As WHc-T77 or WHc-T92 is obviously compatible with WHV infectivity in woodchucks, the sequence context (e.g., other residues in WHc) may compensate for the effect induced by T77 and/or T92 on infectivity, or unknown woodchuck host factor(s) may rescue the WHc T77/T92 infectivity defect.

Our cross-species complementation experiments indicated that WHc or HBc is competent to support intracellular replication of the heterologous virus, in agreement with previous studies [33–36]. Notably, WHc supported much higher packaging efficiency of WHV pgRNA than HBV pgRNA, suggesting specific interactions of WHc preferentially with either the WHV RT and/or WHV pgRNA over the HBV counterparts. WHc-T77E, partially mimicking HBc, actually enhanced, rather than reduced, HBV or WHV pgRNA packaging, while WHc-T92N showed little effect on pgRNA packaging for both viruses. Thus, T77 and T92 are unlikely to account for the enhanced pgRNA packaging by WHc vs. HBc. Also, the much higher WHV pgRNA packaging efficiency by WHc in woodchuck WCH-17 cells than in human HepG2 cells is consistent with host cell modulation of the pgRNA packaging process, e.g., by regulating the HBc/WHc CTD phosphorylation state that modulates pgRNA packaging [67]. Further comparative studies of pgRNA packaging in WHV vs. HBV should help to elucidate the viral and host factors involved in pgRNA packaging, a process still poorly understood but targeted actively for antiviral drug development [67,68]. Alternatively, the apparent enhancement of pgRNA packaging efficiency (i.e., with normalizing to capsid levels) could be due to indirect effects of decreased levels of capsids formed by WHc in case the absolute levels of packed pgRNA stay unchanged. The much lower levels of capsids formed by WHc vs. HBc in woodchuck cells indicate that WHc forms much lower levels of empty capsids than HBc, which may be, at least partially, responsible for the very low secretion levels of empty virions in WHV-infected woodchucks [28], in contrast to the large excess of HBV empty virions in patients [13,15].

One of the most intriguing findings in our study is that WHc supported more efficiently cccDNA formation than HBc in the context of either HBV or WHV and regardless of the host cell line used, suggesting that WHc has a much stronger intrinsic ability than HBc to facilitate intracellular cccDNA amplification, which may account for the higher WHV cccDNA copy numbers in WHV-infected woodchuck hepatocyte than those of HBV cccDNA in HBV-infected chimpanzee or human hepatocytes [41–43]. Although WHc failed to support HBV virion secretion, similar to the HBc-E77T and HBc-E77T/N92T mutants, it still could support 10- to 20-fold higher HBV cccDNA formation efficiency than HBc-E77T and HBc-E77T/N92T, suggesting that an intrinsic property of mature NCs formed by WHc, beyond its inability to support virion secretion, was a dominant factor in supporting efficient cccDNA formation. Similar to certain HBc NTD and linker mutants with enhanced cccDNA amplification ability that we have identified previously [64,69], WHc-containing nucleocapsids failed to protect their rcDNA content against exogenous nuclease digestion. These results suggest that WHc, similar to the above HBc mutants, may enhance intracellular cccDNA amplification via decreased stability/integrity of mature nucleocapsids. The strong enhancing effects on HBV cccDNA by WHc, as compared to HBc, was thus due to a combination of the lack of HBV virion secretion and enhanced nucleocapsid uncoating with WHc. The more dramatic effect of WHc over HBc on cccDNA with the HBV replicon (30-fold difference) than with the WHV replicon (2-6-fold difference) was likely caused by the inability of WHc to complement HBV virion formation. For the WHV replicon, both WHc and HBc failed to support WHV virion production under our cell culture conditions, so the effect of WHc vs. HBc on WHV cccDNA formation was due exclusively to the intrinsic instability of mature nucleocapsids formed by WHc compared to HBc without the contribution from the virion secretion effect. In the HBV-infected human hepatocytes and WHV-infected woodchuck hepatocytes *in vivo*, both HBV and WHV are competent in virion secretion and the difference in cccDNA levels in the two natural infections *in vivo* is thus expected to be influenced only by the intrinsic stability/disassembly efficiency. Consistent with this interpretation, the reported difference in cccDNA levels between WHV and HBV in their naturally infected hosts *in vivo* (20–60 copies for WHV vs. 1–10 copies for HBV) [41] is indeed similar to our observed difference in cccDNA formation supported by WHc vs. HBc when complementing the WHV replicon in cell culture. On the other hand, substitution of T/E at position 77 and of T/N at position 92, however, cannot account for the differential effects of WHc vs. HBc on cccDNA amplification, and future studies are needed to better understand how core sequences control the levels of cccDNA. This also has significant implications for antiviral development as the elimination of this key viral molecule remains the holy grail for an HBV cure.

Our results also indicated that WHV capsids could not interact with HBV envelope proteins in supporting the secretion of chimeric virions. Recombinant WHV capsid shares a high structural similarity with the HBV capsid [19]. A short linear sequence in the PreS1 domain (i.e., matrix domain or MD) is thought to be required for interaction with the HBV capsid for virion morphogenesis [39]. Interestingly, this MD is conserved between HBV and WHV [39]. Specifically, twelve residues on the HBV capsid surface (i.e., S17, F18, L60, L95, K96, L100, F122, I126, R127, N136, A137, and I139) involved in forming the matrix binding domain (MBD) are thought to be important for envelope interactions [32,65]. Most of these MBD residues are also conserved between HBc and WHc, but S17, L60, L100, in HBc are N17, V60, and S100, respectively, in WHc, which might contribute to the inability of WHc to interact with the HBV envelope. Indeed, HBc-L60V has been reported to reduce HBV virion secretion [70]. In addition to these residues, our results here indicate that E77 in HBc is crucial for HBV envelope interaction. However, the WHc-T77E and WHc-T92N substitutions failed to rescue WHV virion secretion with the HBV envelope proteins. We have recently shown that the HBc linker sequence also plays a critical role in supporting virion secretion [64,71] but this appears less likely as the linker sequence is almost entirely conserved in WHc (with only one conservative change of V149I; **Fig 1A**). Thus, future studies on WHc and HBc cross-species complementation would help to further identify the determinants critical for capsid-envelope interactions during virion morphogenesis.

In contrast to all known avihepadnaviruses, which have (or are predicted to have) glycosylated precore proteins, WHV, ASHV, GSHV, DCHBV, MDHBV, and EqHBV are the only known orthohepadnaviruses that have N-glycosylation motifs in their precore/core genes. Phylogenetic analysis suggests that modern day hepadnaviruses are derived from an ancient, non-enveloped virus (i.e., nackednaviruses) [72]. However, no N-glycosylation motifs are present in the core ORFs of nackednaviruses (**Fig 9B**), suggesting that the N-glycosylation sequons most likely were gained later during the evolution of avihepadnaviruses and orthohepadnaviruses, which are the only hepadnaviruses that acquired the signal peptide sequences necessary for e antigen secretion [44]. Considering the important role of e antigen in immunomodulation and viral persistence [73], acquisition of e antigen (precore) glycosylation, which, as we have shown here, enhances the levels of HBeAg and WHeAg (as well as the levels of HBV and WHV PreC, another secreted precore product), was likely selected positively during hepadnavirus evolution. In addition, as maternal HBeAg is thought to cross the placenta and to induce T cell tolerance to HBV *in utero* [23,24], it will be interesting to determine if and how N-glycosylation of e antigen affects placental transfer or any other potential host interactions of e antigen. On the other hand, the essential replication functions of the core protein needed to be maintained during the evolution of the overlapping precore gene. As the precore gene likely evolved after the emergence of the envelope proteins, as suggested by the fact that only avihepadnaviruses and orthohepadnaviruses possess the precore gene [44], and considering that the emergence of the viral envelope proteins is thought to have co-evolved with the host species [72], the essential role of HBc-E77 in supporting interactions with the envelope proteins for virion secretion would have thus precluded a E77T substitution required for acquisition of the N-glycosylation sequon at this location of the overlapping precore gene for the primate hepadnaviruses. As we have shown, the impairment of infectivity by the E77T or N92T substitution in HBc would have further prevented the acquisition of HBV precore N-glycosylation at both positions (**Fig 9B**). The alternative possibility, which we consider less likely but can’t exclude, is that the N-glycosylation sequons had evolved in a common ancestor to avihepadnaviruses and orthohepadnaviruses, subsequent to nackdnaviruses, but were lost subsequently in the primate hepadnaviruses, consistent with the later appearance of primates than birds and rodents. Overlapping genes, widely present in compact viral genomes, arise as a result of overprinting, a process inducing the expression of a *de novo* protein from pre-existing nucleotide sequences [74]. Different from other viruses and other hepadnaviral proteins which use overlapping but *alternative* reading frames to encode different gene products, the hepadnavirus precore gene employs the entire coding sequences of the core gene, in the *same* reading frame. This arrangement has likely exerted stringent constraints on the evolution of both genes, as uncovered here, and provides an excellent example to study the evolution of overlapping genes.

## Materials and methods

### Plasmids

The WT HBV replication-competent plasmid replicon pCIΔA-HBV-HBc-WT (genotype D) was described previously [63]. Full-length HBV replicons gaining the N-glycosylation motif(s), pCIΔA-HBV-HBc-E77T, pCIΔA-HBV-HBc-N92T, and pCIΔA-HBV-HBc-E77T/N92T were constructed by substituting the E77 and N92 residues on the HBV precore/core with the corresponding residues T77 and T92 on the WHV precore/core by PCR-directed mutagenesis. The WT WHV strain 8 (WHV8) replicon plasmid pucCMVWHV, directing WHV pgRNA under the control of immediate early cytomegalovirus promoter, was constructed and described in early studies [60,75]. Full-length WHV replicons losing the N-glycosylation motif(s), pucCMVWHV-WHc-T77E, pucCMVWHV-WHc-T92N, and pucCMVWHV-WHc-T77E/T92N were constructed by substituting the T77 and T92 residues on the WHV precore/core with the corresponding residues E77 or N92 on the HBV precore/core. pSVHBV1.5Core^-^ (genotype A) (a gift from Volker Bruss, Technical University of Munich) and pCIΔA-HBV-HBc-C^-^ (genotype D) are HBV replicon constructs defective in HBc expression but support HBV replication upon complementation with HBc expressing constructs [32,64]. pucCMVWHV-C^-^ was generated by PCR-directed substitution of the core ORF start codon (ATG) to GTG on pucCMVWHV to silence WHc expressions.

The HBc expression constructs including pCI-HBc, pCI-HBc-E77T, pCI-HBc-N92T, and pCI-E77T/N92T were constructed by subcloning the HBc gene from the full-length HBV replicon and inserting into the pCI vector (Promega) as described [16]. Similarly, HBV precore expression constructs, pCI-HPC-WT, pCI-HPC-E77T, pCI-HPC-N92T, and pCI-HPC-E77T/N92T; WHc expression constructs, pCI-WHc, pCI-WHc-T77E, pCI-WHc-T92N, and pCI-WHc-T77E/T92N; and WHV precore expression constructs, pCI-WPC, pCI-WPC-T77E, pCI-WPC-T92N, and pCI-WPC-T77E/T92N were generated [16,28].

### Cell cultures

Human hepatoma cell lines HepG2, Huh7, HepG2-huNTCP, and the woodchuck hepatoma cell line WC3 (a gift from Haitao Guo, University of Pittsburgh) and WCH-17 (CRL-2082, ATCC) were cultured in Dulbecco’s modified Eagle’s medium (DMEM)-F12 supplemented with 10% fetal bovine serum (FBS) (Hyclone) and 50 μg/ml of penicillin-streptomycin as described previously [16,28,37,63].

### Transient transfection

Transient transfections of HepG2, Huh7, WC3, and WCH-17 cells were performed as previously described [16,37,63,76]. HepG2, WC3, and WCH-17 cells seeded in 60-mm dishes were transfected with a total of 4 μg of plasmid using X-tremeGENE HP DNA Transfection Reagent (Roche). Huh7 cells seeded in 60-mm dishes were also transfected with a total of 4 μg of plasmid using FuGENE6 Transfection Reagent (Promega). A 1:1 mass ratio of each plasmid was used to perform a two plasmid co-transfection experiment [14,16,76]. All transfection experiments were repeated between two and five times.

### Immunoblot analysis

Concentrated cell culture supernatant and cell lysates were separated using 12.5% sodium dodecyl sulfate (SDS)-polyacrylamide gel electrophoresis (PAGE) as described previously [13,16,28]. The mouse monoclonal antibodies (mAb) clone T2221 (Cat no. 2AHC24) specific for HBV precore/core NTD and clones C33 (Cat no. 2ZC33) and 19C18 (Cat no. 2Z19C18) that are predicted to recognize equally both the HBV and WHV precore/core NTD (**Fig 1**) were purchased from Tokyo Future Style [77]. The polyclonal rabbit antibody against HBsAg (Cat no. 1811) (Virostat, Portland, ME, USA) was used for the detection of surface proteins. The mouse anti-precore mAb, 1A11, specific for WHV or HBV precore-related proteins was a gift from Peter Revill (VIDRL, Australia) [16,28,78].

### Analysis of viral DNA from transfected cells

The HBV core DNAs were released from the cytoplasmic lysate containing viral nucleocapsids by disrupting capsids via treatment with 0.5% SDS and subsequent incubation with 0.6 mg/ml Protease K (Invitrogen) at 37°C for 1 hour [63,64]. Hirt extraction was employed for extracting protein-free (PF) DNAs [63,64]. Dpn I digestion was used for the removal of input plasmids from HBV PF-DNA. Dpn I-treated DNA was further digested with Exonucleases I and III (Exo I and Exo III) to remove all DNAs with free 3’ ends but preserve closed circular DNAs, both double-stranded and single-stranded, as described previously [37,63,79]. Viral DNAs were resolved on a 1.2% agarose gel and detected by a ^32^P-labeled HBV or WHV DNA probe.

### Native agarose gel electrophoresis (NAGE) for analyzing capsid assembly and pgRNA packaging

To analyze the levels of assembled capsids and the amount of pgRNA packaged inside capsids, cytoplasmic NP40 lysate was resolved by 1% native agarose gel as described previously [13]. Following the transfer of viral particles from the gel to a nitrocellulose membrane, a ^32^P-labelled HBV or WHV anti-sense RNA riboprobe was used to detect encapsidated HBV or WHV pgRNA, respectively. Subsequently, the capsids were detected by mouse monoclonal anti-HBc (T2221) or anti-HBc/WHc (C33) antibody as described [13,52,76].

### Assay for virion secretion

Cell culture supernatant was precipitated with polyethylene glycol (PEG) 8000 [63]. Concentrated supernatant (by 50 times) was digested with DNase I to remove plasmid DNAs and resolved by 1% native agarose gel as described previously [13,64]. Following the transfer of viral particles from the gel to a nitrocellulose membrane, a ^32^P-labelled HBV or WHV DNA probe was used to detect HBV or WHV DNA within viral particles. Subsequently, the capsid or surface proteins were detected by the above core- or surface-specific antibody, as described previously [13,52,76].

### HBV inoculum quantification

Culture supernatant of transfected Huh7 cells was collected at days 5, 7, and 9 post-transfection and concentrated by PEG 8000 as above (by 100 times), which was used as the inoculum for infection. To determine the inoculum viral titer, the concentrated supernatant was resolved by agarose gel electrophoresis and transferred to nitrocellulose membrane, and HBV DNA associated with virions and naked capsids was detected by a ^32^P-labeled HBV DNA probe. Serial dilutions of the HBV replicon plasmid (pCIΔA-HBV-HBc-WT) served as quantification standards. Only the virion-associated DNA was quantified in the calculation of the HBV genome equivalent used for infection.

### HBV infection

HBV infection in HepG2-huNTCP cells was carried out as described [37,63,64]. Briefly, HepG2-huNTCP cells were plated in collagen I-coated 35-mm culture dishes (Corning). When the cells reached 100% confluence, they were infected with WT or mutant HBV inoculum derived from transfected Huh7 cells, at a multiplicity of infection (MOI) of ca. 200 or at the indicated genome of equivalent per cell (GE/cell) in the presence of 2% DMSO and 4% PEG 8000. After 16 hours, the HBV inoculum was removed and fresh medium containing 2% DMSO was added. At 4 days or 9 days post-infection, the cells were harvested and subjected to Hirt extraction. PF-DNA was further analyzed by Southern blot analysis as described above.

### Phylogenetic analysis

The genomic DNA sequences of Sheldgoose HBV (SheldGHBV, NC_005890), DHBV (NC_001344), DHBV (AY494851), snow goose HBV (SGHBV, NC_005950), Ross’s goose HBV (RGHBV, NC_005888), crane HBV (CCHBV, AJ441111), parrot HBV (PHBV, NC_016561), stork HBV (STHBV, AJ251934), heron HBV (HHBV, NC_001486), tent making bat HBV (TBHBV, KC790378), long fingered bat HBV (LBHBV, JX941466), roundleaf bat HBV (RBHBV, KC790373), horseshoe bat (HBHBV, KC790377), domestic cat HBV (DCHBV, NC_040719), Maxwell’s duiker HBV (MDHBV, MK620908), equid HBV (EgHBV, MT134279), GSHV (K02715), WHV (M18752), ASHV (Q64896), woolly monkey HBV (WMHBV, AF046996), capuchin monkey HBV (CMHBV, KY703886), human HBV genotype A (HBV GtA, AB116076), human HBV genotype D (HBV GtD, AB104711), gibbon HBV (GiHBV, AJ131572), gorilla HBV (GoHBV, AJ131567), orangutan HBV (OrHBV, AF193864), and chimpanzee HBV (ChHBV, FJ798099) were downloaded from the NCBI Nucleotide database (https://www.ncbi.nlm.nih.gov/nuccore). The hepadnaviral precore/core gene sequences were aligned using Clustal Omega and the phylogenetic trees of genomes were constructed using Maximum-Likelihood method implemented in the PhyML program (v3.1/3.0 aLRT) [80].

### Statistical analysis

Protein and DNA signals from western blot analysis and Southern blot analysis were detected by the Image Lab system 6.0.1 (Bio-Rad) or Sapphire Biomolecular Imager (Azure Biosystems), respectively. Protein and DNA signals were quantified using the Image Lab system 6.0.1 (Bio-Rad), as described [16,64]. Data were analyzed by using Prism 7.0 (GraphPad). Student’s t test, two-tailed and unpaired, was used to compare two datasets, and the data is shown as mean ± standard deviation (mean ± SD). *p* < 0.05 was considered to be statistically significant.

## Supporting information

S1 Fig All known avihepadnaviruses and some orthohepadnaviruses have N-glycosylation sequons in their precore/core gene products. **(A)** Genomic-length maximum-likelihood phylogeny of all identified *Avihepadnavirus* and *Orthohepadnavirus* sequences. The hepadnaviruses with putative N-glycosylation sequons in their precore/core gene products are highlighted in red. **(B)** Amino acid sequence alignment of the avihepadnaviruses precore/core proteins. One or two identified or putative N-glycosylation sequons are highlighted in the red boxes. **(C)** Amino acid sequence alignment of the orthohepadnaviruses precore/core proteins. One or two identified or putative N-glycosylation sequons are highlighted in the red boxes. Numberings start from the first methionine of the core ORF.
(TIF)

S2 Fig WHV precore N-glycosylation enhanced the secretion of the precore gene products in woodchuck cells. Immunoblot analysis of WHV precore gene products in the culture supernatant of WC3 cells transfected with WHV precore constructs. The supernatants were concentrated by ultrafiltration and treated with PNGase F or not and resolved by regular SDS-PAGE, followed by immunoblotting with mAb 1A11. WHV core-transfected cell culture supernatant served as the control for the background bands. *, cross-reactive background bands.
(TIF)

S3 Fig Effects of eliminating N-glycosylation sequons on WHc functions. The WHV replicon construct expressing the T77E, T92N, or T77E/T92N WHc mutant, or WT WHc was transfected into WC3 or Huh7 cells. **(A)** The assembled capsids (top) and packaged pgRNA (middle) were detected by the C33 anti-HBc/WHc mAb and anti-sense WHV RNA probe, respectively, following the resolution of cytoplasmic lysates from the transfected WC3 cells by NAGE and transfer to nitrocellulose membrane. Levels of WHc proteins (bottom) were measured by western blot using 19C18 anti-HBc/WHc mAb after SDS-PAGE. Capsid assembly efficiency **(B)** was determined by normalizing the levels of capsids to those of total WHc protein, and pgRNA packaging efficiency **(C)** was determined by normalizing the levels of pgRNA to those of capsids, with the efficiencies of WT WHc set to 1.0. **(D)-(F)** Cytoplasmic lysates from WHV replicon transfected Huh7 cells were analyzed for capsid assembly and pgRNA packaging, as described for WC3 cells. **(G)** WHV core DNA was released from the NCs of cytoplasmic lysate by SDS-proteinase K treatment and detected by Southern blot analysis. Due to the non-detectable WHV rcDNA in transfected WC3 cells, (rcDNA) denotes for the expected position of WHV rcDNA. WHV PF-DNA was extracted from the transfected WC3 cells by Hirt extraction. The extracted DNA was treated with Dpn I plus the exonucleases I and III (Exo I & III) to remove all DNA with free 3’ ends. The ssDNA synthesis efficiency **(H)** was determined by normalizing the levels of ssDNA to those of pgRNA in (**A**) with the efficiency of WT WHc set to 1.0. Similarly, core DNA and PF-DNA from WHV replicon transfected Huh7 cells were analyzed **(I)** and the ssDNA synthesis efficiency **(J)** was determined by normalizing the levels of ssDNA to those of pgRNA in (**D**) with the efficiency of WT WHc set to 1.0. The cccDNA formation efficiency **(K)** was determined by normalizing the levels of cccDNA in **(I)** to those of rcDNA with the efficiency from WT WHc set to 1.0. Data is shown as mean ± SD. Two-tailed unpaired Student’s t test was used to compare the difference of each data set versus WT WHc (*, *p* < 0.05; **, *p* <0.01; ***, *p* < 0.001). Ca, capsid; pgRNA, pregenomic RNA; WHc, WHV core protein; ssDNA, single-strand DNA; rcDNA, relaxed circular DNA; cccDNA, covalently closed circular DNA; cM-DNA, closed minus strand DNA.
(TIF)

S4 Fig Analysis of WHV virion secretion from the WHc N-glycosylation mutants. The WHV replicon construct expressing the WT WHc **(A**) or the T77E **(B)**, T92N **(C)**, or T77E/T92N **(D)** WHc mutants was transfected into HepG2 cells. Cell culture supernatant from WHV-transfected HepG2 cells were collected at day 14 post-transfection and fractionated by CsCl gradient ultracentrifugation. Indicated fractions (fractions 14 to 22) were resolved by NAGE and detected with a WHV DNA probe. Fraction 17 is known to contain the WHV virion peak at a density of 1.258 g/cm^3^. NC, nucleocapsids.
(TIF)

S5 Fig Effects of introducing N-glycosylation sequons on HBc functions. **(A)** Cell culture supernatant from HBV replicon transfected Huh7 cells was collected at day 5 post-transfection. Concentrated (50x) supernatant was resolved by agarose gel electrophoresis and transferred to nitrocellulose membrane. HBV DNA and capsids associated with virions and naked capsids, and envelope proteins in virions and subviral particles were detected sequentially using a ^32^P-labeled HBV DNA probe, the anti-HBc T2221 mAb, and anti-HBs polyclonal antibody, respectively, on the same membrane. **(B)** Culture supernatant of transfected Huh7 cells was collected at days 5, 7, and 9 post-transfection and concentrated (100X). The collected and concentrated supernatant was analyzed as in **A**. **(C)** Infection of HepG2-huNTCP cells with the N92T mutant virus at increasing dosage (MOI = 200, 400, 800, or 1600 GE/cell). HBV PF-DNA was extracted from infected cells at 9 days post-infection and analyzed by Southern blot analysis. Ca, capsid; V, virion; HBs, HBV surface antigen; PF-rcDNA, protein-free rcDNA; cccDNA, covalently closed circular DNA.
(TIF)

S6 Fig Ability of WT and mutant HBc or WHc to support HBV replication in the *trans*-complementation assay. The HBV replicon construct that is defective in HBc expression was co-transfected with WT or mutant HBc or WHc expression construct into WCH-17 cells. **(A)** The assembled capsids (top) and packaged pgRNA (middle) were detected by the C33 anti-HBc/WHc mAb and anti-sense HBV RNA probe, respectively, following the resolution of cytoplasmic lysates by NAGE and transfer to nitrocellulose membrane. Levels of HBc or WHc proteins (bottom) were measured by western blot using the 19C18 anti-HBc/WHc mAb after SDS-PAGE. Capsid assembly efficiency **(B)** was determined by normalizing the levels of capsids to those of total HBc/WHc protein, and pgRNA packaging efficiency **(C)** was determined by normalizing the levels of pgRNA to those of capsids, with the efficiencies of WT HBc set to 1.0. **(D)** core DNA was released from the NCs of cytoplasmic lysate by SDS-proteinase K treatment and detected by Southern blot analysis. **(E)** HBV PF-DNA was isolated from the transfected cells by the Hirt extraction method. The extracted DNA was treated with Dpn I plus Exo I & III to remove all DNA with free 3’ ends. **(F)** The cccDNA formation efficiency was calculated by normalizing the levels of cccDNA to those of rcDNA, with the efficiency from WT HBc set to 1.0. **(G)** Summary of all parameters of HBV replication in HepG2 (shown in **Fig 6**) and WCH-17 cells. Increased parameters compared to HBc-WT were marked in red while decreased parameters compared to HBc-WT were marked in blue. Data is shown as mean ± SD. Two-tailed unpaired Student’s t test was used to compare the difference of each dataset versus WT HBc (*, *p* < 0.05; **, *p* <0.01; ***, *p* < 0.001). Ca, capsid; pgRNA, pregenomic RNA; HBc, HBV core protein; WHc, WHV core protein; ssDNA, single-strand DNA; rcDNA, relaxed circular DNA; cccDNA, covalently closed circular DNA; cM-DNA, closed minus strand DNA.
(TIF)

S7 Fig Ability of WT and mutant HBc or WHc to support WHV replication in the *trans*-complementation assay. The WHV replicon construct that is defective in WHc expression was co-transfected with WT or mutant HBc or WHc expression construct into HepG2 cells. **(A)** The assembled capsids (top) and packaged pgRNA (middle) were detected by the C33 anti-HBc/WHc mAb and anti-sense HBV RNA probe, respectively, following the resolution of cytoplasmic lysates by NAGE and transfer to nitrocellulose membrane. Levels of HBc or WHc proteins (bottom) were measured by western blot using 19C18 anti-HBc/WHc mAb after SDS-PAGE. Capsid assembly efficiency **(B)** was determined by normalizing the levels of capsids to those of total HBc/WHc protein, and pgRNA packaging efficiency **(C)** was determined by normalizing the levels of pgRNA to those of capsids, with the efficiencies of WT HBc set to 1.0. **(D)** core DNA was released from the NCs of cytoplasmic lysate by SDS-proteinase K treatment and detected by Southern blot analysis. **(E)** HBV PF-DNA was isolated from the transfected cells by the Hirt extraction method. The extracted DNA was treated with Dpn I plus Exo I & III to remove all DNA with free 3’ ends. **(F)** The cccDNA formation efficiency was calculated by normalizing the levels of cccDNA to those of rcDNA, with the efficiency from WT HBc set to 1.0. **(G)** Summary of all parameters of WHV replication in HepG2 and WCH-17 cells (shown in **Fig 6**). Increased parameters compared to HBc-WT were marked in red while decreased parameters compared to HBc-WT were marked in blue. Data is shown as mean ± SD. Two-tailed unpaired Student’s t test was used to compare the difference of each data set versus WT HBc (*, *p* < 0.05; **, *p* <0.01; ***, *p* < 0.001). Ca, capsid; pgRNA, pregenomic RNA; HBc, HBV core protein; WHc, WHV core protein; ssDNA, single-strand DNA; rcDNA, relaxed circular DNA; cccDNA, covalently closed circular DNA; cM-DNA, closed minus strand DNA.
(TIF)

S8 Fig Effects of HBc or WHc on protecting the rcDNA content inside NCs. Core DNA released from the cytoplasmic lysate of WT or mutant HBc or WHc co-transfected with HBV-C(-) from WCH-17 cells **(A)** or with WHV-C(-) from HepG2 cells **(B)**, with or without prior Turbo DNase digestion, was detected by Southern blot analysis. The rcDNA signals obtained after Turbo DNase treatment is indicated by the dashed, red box for comparison to rcDNA signals present without nuclease treatment. ssDNA, single strand DNA; rcDNA, relaxed circular DNA.
(TIF)
