# Supplementary figures and images for: Constrained evolution of overlapping genes in viral host adaptation: Acquisition of glycosylation motifs in hepadnaviral precore/core genes
Source: PLoS Pathog. 2022 Jul 28;18(7):e1010739. doi: 10.1371/journal.ppat.1010739 (PMC9362955; doi:10.1371/journal.ppat.1010739)

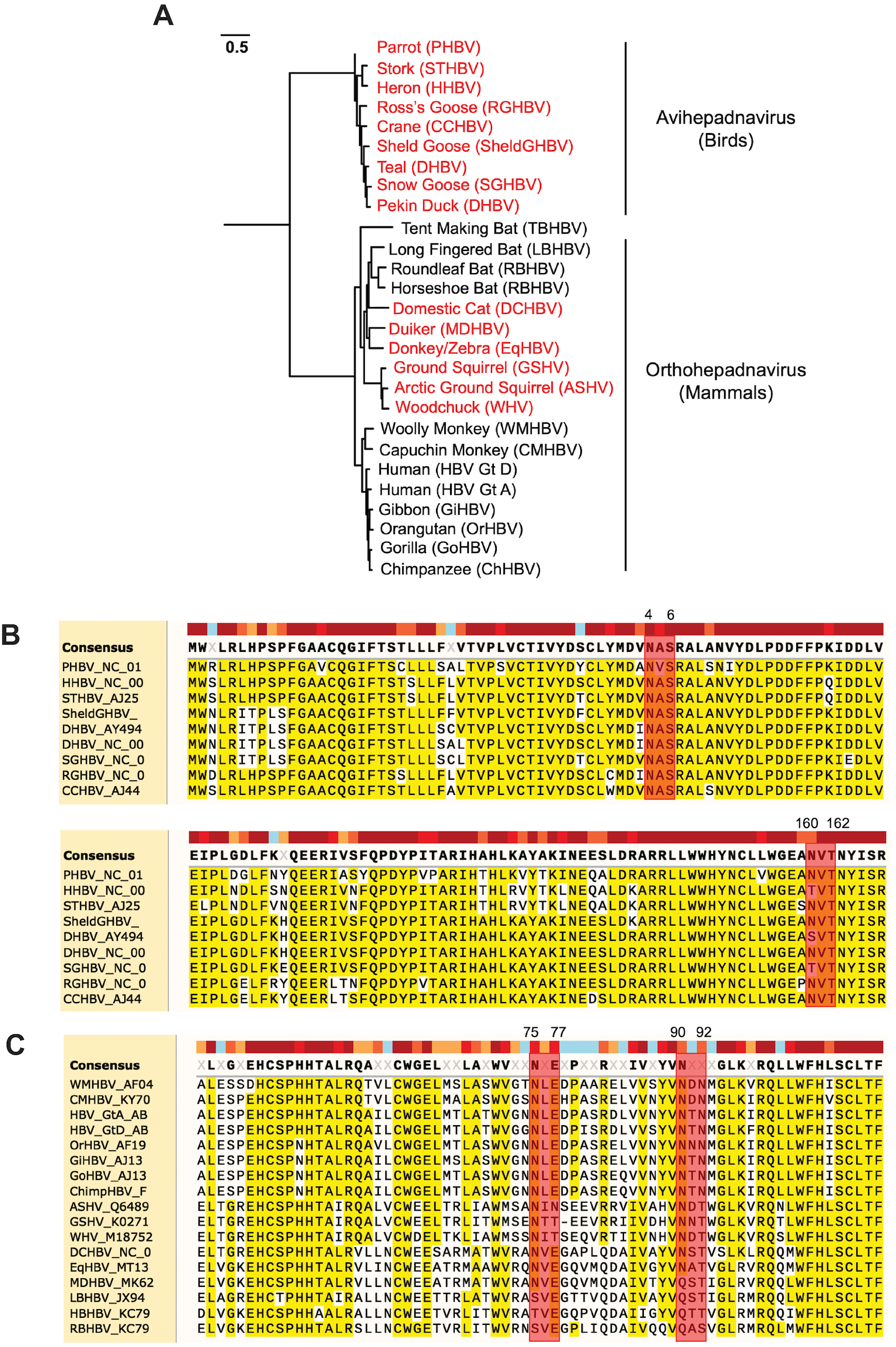

Supplement: S1 Fig — (A) Genomic-length maximum-likelihood phylogeny of all identified Avihepadnavirus and Orthohepadnavirus sequences. The hepadnaviruses with putative N-glycosylation sequons in their precore/core gene products are highlighted in red. (B) Amino acid sequence alignment of the avihepadnaviruses precore/core proteins. One or two identified or putative N-glycosylation sequons are highlighted in the red boxes. (C) Amino acid sequence alignment of the orthohepadnaviruses precore/core proteins. One or two identified or putative N-glycosylation sequons are highlighted in the red boxes. Numberings start from the first methionine of the core ORF. (TIF) [file ppat.1010739.s001.tif]

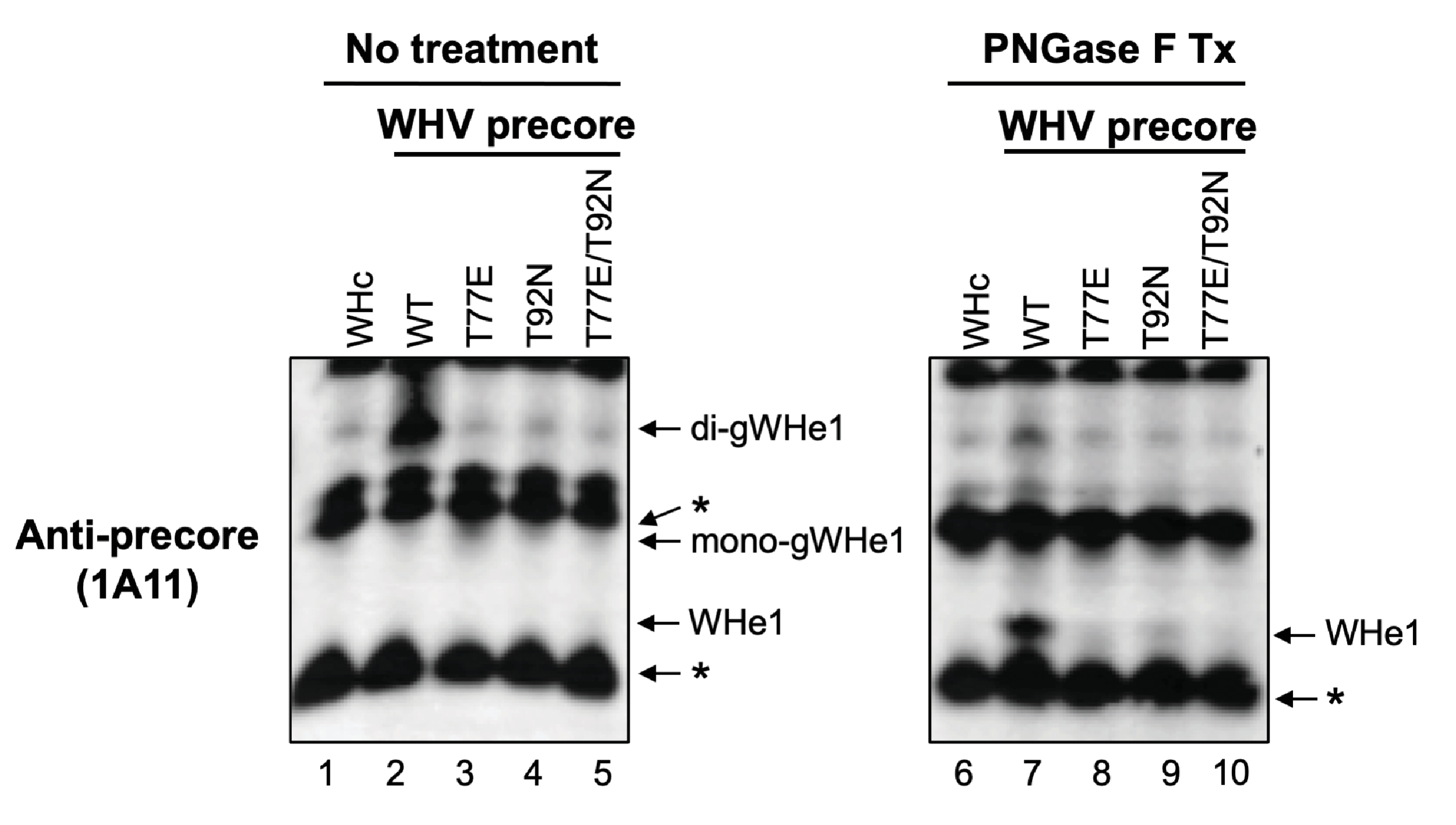

Supplement: S2 Fig — Immunoblot analysis of WHV precore gene products in the culture supernatant of WC3 cells transfected with WHV precore constructs. The supernatants were concentrated by ultrafiltration and treated with PNGase F or not and resolved by regular SDS-PAGE, followed by immunoblotting with mAb 1A11. WHV core-transfected cell culture supernatant served as the control for the background bands. *, cross-reactive background bands. (TIF) [file ppat.1010739.s002.tif]

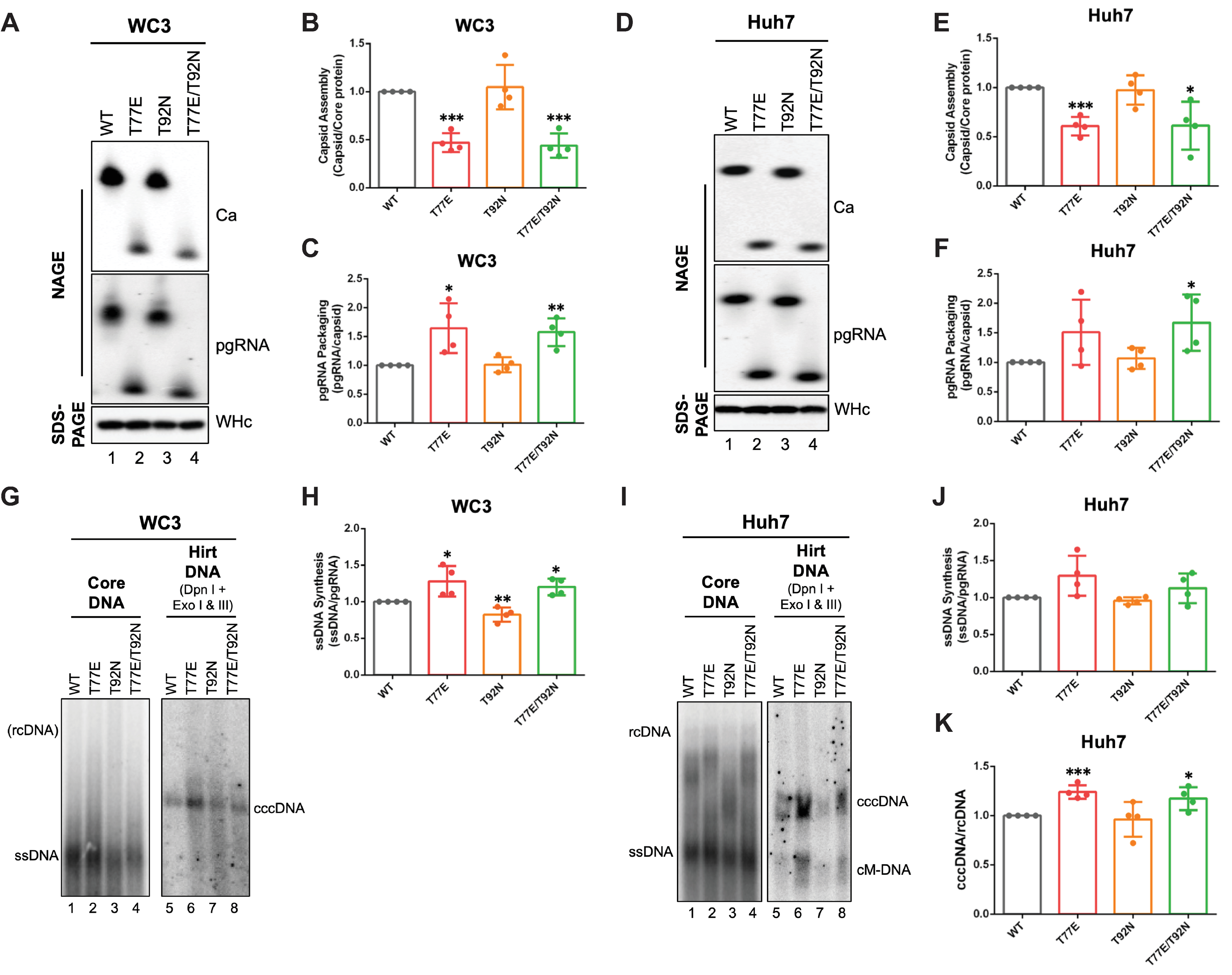

Supplement: S3 Fig — The WHV replicon construct expressing the T77E, T92N, or T77E/T92N WHc mutant, or WT WHc was transfected into WC3 or Huh7 cells. (A) The assembled capsids (top) and packaged pgRNA (middle) were detected by the C33 anti-HBc/WHc mAb and anti-sense WHV RNA probe, respectively, following the resolution of cytoplasmic lysates from the transfected WC3 cells by NAGE and transfer to nitrocellulose membrane. Levels of WHc proteins (bottom) were measured by western blot using 19C18 anti-HBc/WHc mAb after SDS-PAGE. Capsid assembly efficiency (B) was determined by normalizing the levels of capsids to those of total WHc protein, and pgRNA packaging efficiency (C) was determined by normalizing the levels of pgRNA to those of capsids, with the efficiencies of WT WHc set to 1.0. (D)-(F) Cytoplasmic lysates from WHV replicon transfected Huh7 cells were analyzed for capsid assembly and pgRNA packaging, as described for WC3 cells. (G) WHV core DNA was released from the NCs of cytoplasmic lysate by SDS-proteinase K treatment and detected by Southern blot analysis. Due to the non-detectable WHV rcDNA in transfected WC3 cells, (rcDNA) denotes for the expected position of WHV rcDNA. WHV PF-DNA was extracted from the transfected WC3 cells by Hirt extraction. The extracted DNA was treated with Dpn I plus the exonucleases I and III (Exo I & III) to remove all DNA with free 3’ ends. The ssDNA synthesis efficiency (H) was determined by normalizing the levels of ssDNA to those of pgRNA in (A) with the efficiency of WT WHc set to 1.0. Similarly, core DNA and PF-DNA from WHV replicon transfected Huh7 cells were analyzed (I) and the ssDNA synthesis efficiency (J) was determined by normalizing the levels of ssDNA to those of pgRNA in (D) with the efficiency of WT WHc set to 1.0. The cccDNA formation efficiency (K) was determined by normalizing the levels of cccDNA in (I) to those of rcDNA with the efficiency from WT WHc set to 1.0. Data is shown as mean ± SD. Two-tailed unpaired Student’s t test wa [file ppat.1010739.s003.tif]

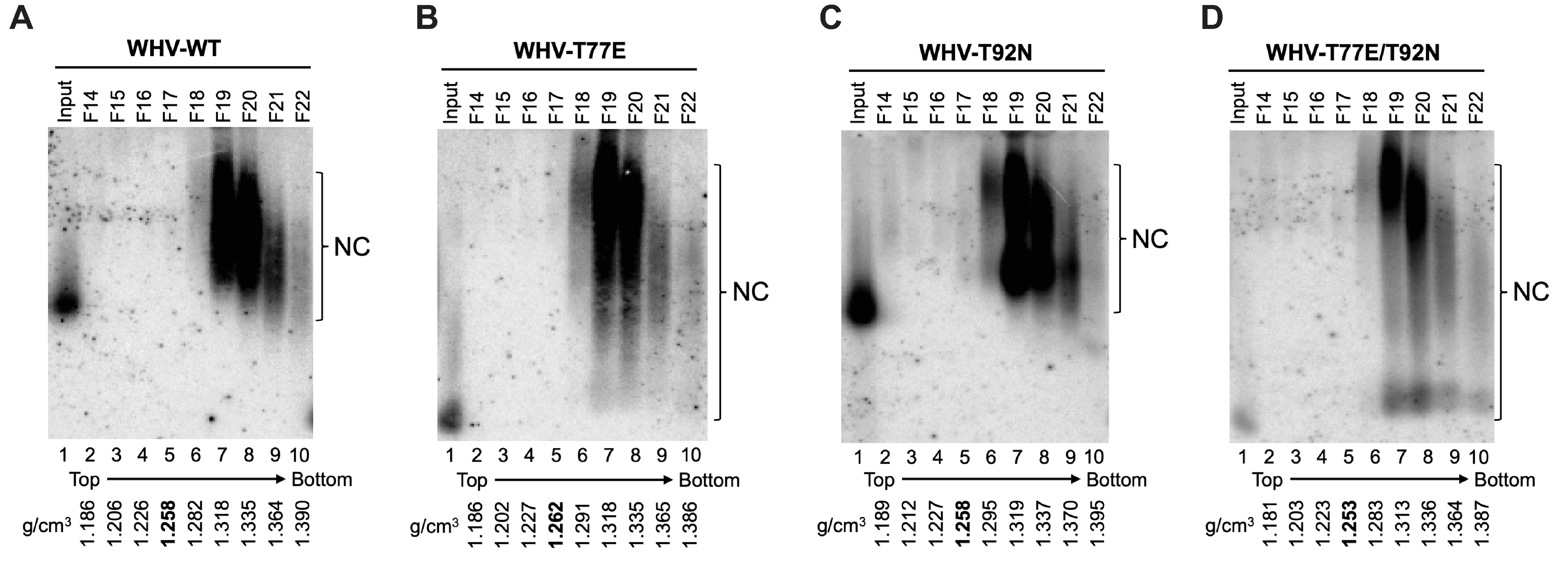

Supplement: S4 Fig — The WHV replicon construct expressing the WT WHc (A) or the T77E (B), T92N (C), or T77E/T92N (D) WHc mutants was transfected into HepG2 cells. Cell culture supernatant from WHV-transfected HepG2 cells were collected at day 14 post-transfection and fractionated by CsCl gradient ultracentrifugation. Indicated fractions (fractions 14 to 22) were resolved by NAGE and detected with a WHV DNA probe. Fraction 17 is known to contain the WHV virion peak at a density of 1.258 g/cm3. NC, nucleocapsids. (TIF) [file ppat.1010739.s004.tif]

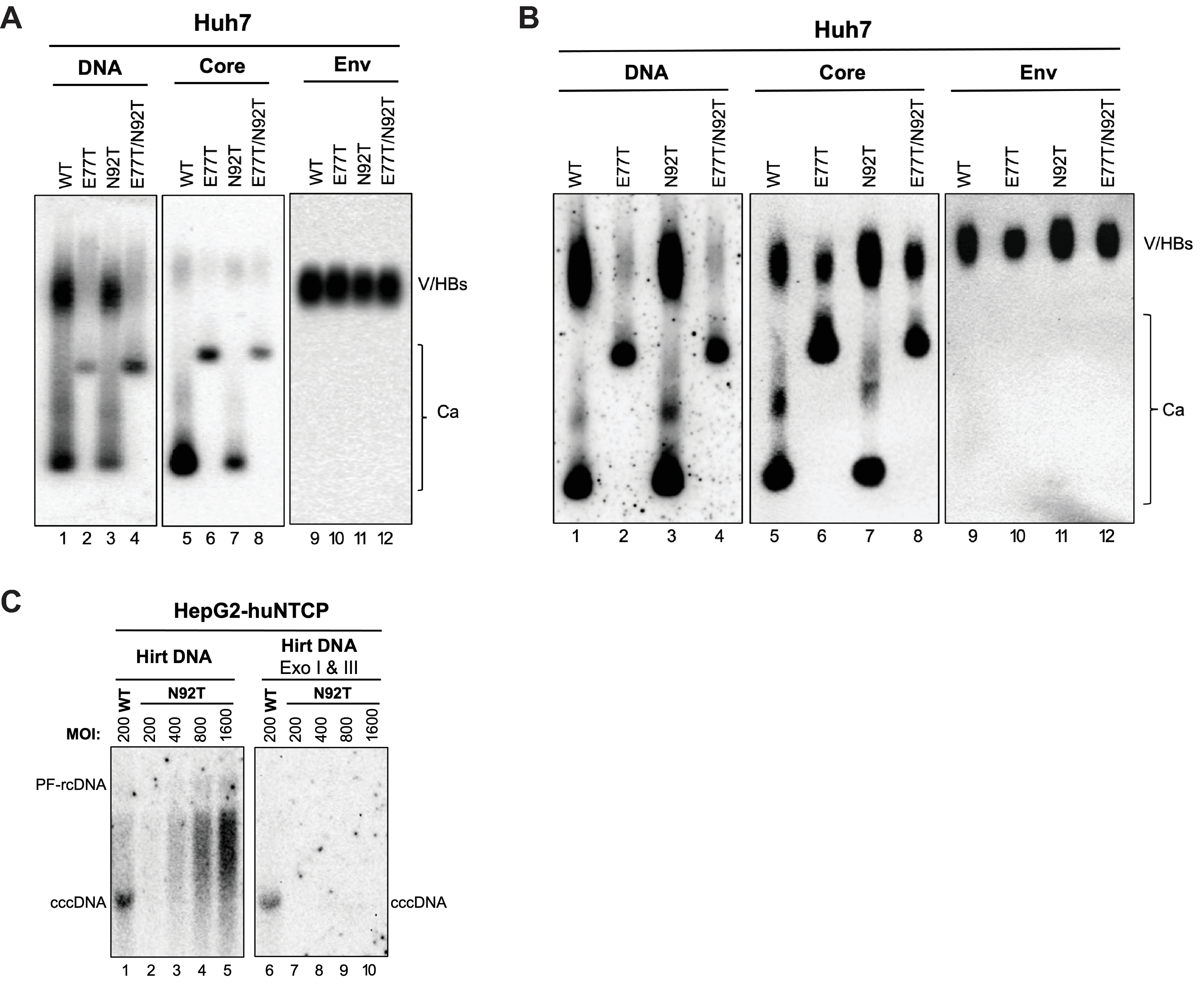

Supplement: S5 Fig — (A) Cell culture supernatant from HBV replicon transfected Huh7 cells was collected at day 5 post-transfection. Concentrated (50x) supernatant was resolved by agarose gel electrophoresis and transferred to nitrocellulose membrane. HBV DNA and capsids associated with virions and naked capsids, and envelope proteins in virions and subviral particles were detected sequentially using a 32P-labeled HBV DNA probe, the anti-HBc T2221 mAb, and anti-HBs polyclonal antibody, respectively, on the same membrane. (B) Culture supernatant of transfected Huh7 cells was collected at days 5, 7, and 9 post-transfection and concentrated (100X). The collected and concentrated supernatant was analyzed as in A. (C) Infection of HepG2-huNTCP cells with the N92T mutant virus at increasing dosage (MOI = 200, 400, 800, or 1600 GE/cell). HBV PF-DNA was extracted from infected cells at 9 days post-infection and analyzed by Southern blot analysis. Ca, capsid; V, virion; HBs, HBV surface antigen; PF-rcDNA, protein-free rcDNA; cccDNA, covalently closed circular DNA. (TIF) [file ppat.1010739.s005.tif]

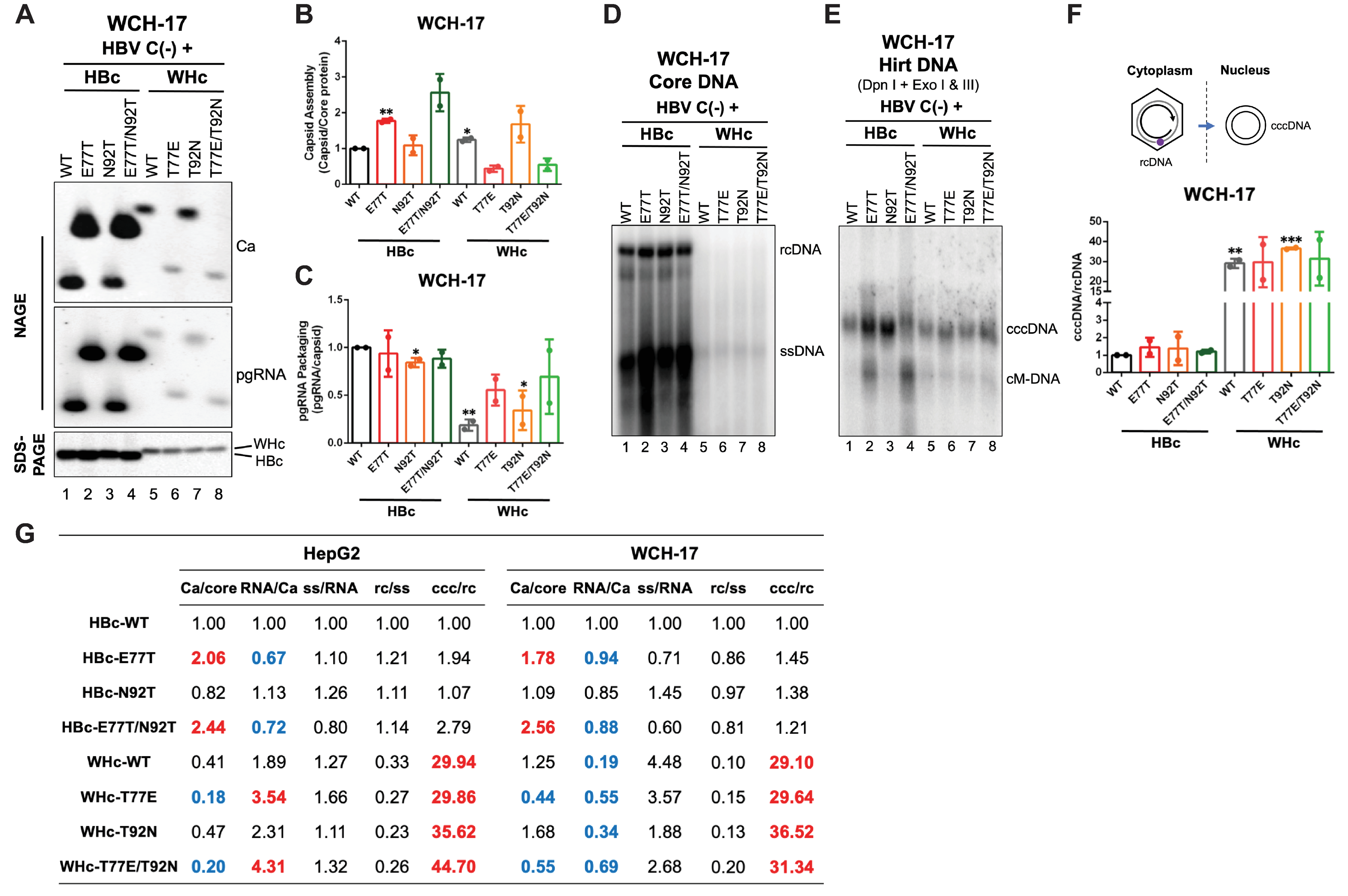

Supplement: S6 Fig — The HBV replicon construct that is defective in HBc expression was co-transfected with WT or mutant HBc or WHc expression construct into WCH-17 cells. (A) The assembled capsids (top) and packaged pgRNA (middle) were detected by the C33 anti-HBc/WHc mAb and anti-sense HBV RNA probe, respectively, following the resolution of cytoplasmic lysates by NAGE and transfer to nitrocellulose membrane. Levels of HBc or WHc proteins (bottom) were measured by western blot using the 19C18 anti-HBc/WHc mAb after SDS-PAGE. Capsid assembly efficiency (B) was determined by normalizing the levels of capsids to those of total HBc/WHc protein, and pgRNA packaging efficiency (C) was determined by normalizing the levels of pgRNA to those of capsids, with the efficiencies of WT HBc set to 1.0. (D) core DNA was released from the NCs of cytoplasmic lysate by SDS-proteinase K treatment and detected by Southern blot analysis. (E) HBV PF-DNA was isolated from the transfected cells by the Hirt extraction method. The extracted DNA was treated with Dpn I plus Exo I & III to remove all DNA with free 3’ ends. (F) The cccDNA formation efficiency was calculated by normalizing the levels of cccDNA to those of rcDNA, with the efficiency from WT HBc set to 1.0. (G) Summary of all parameters of HBV replication in HepG2 (shown in Fig 6) and WCH-17 cells. Increased parameters compared to HBc-WT were marked in red while decreased parameters compared to HBc-WT were marked in blue. Data is shown as mean ± SD. Two-tailed unpaired Student’s t test was used to compare the difference of each dataset versus WT HBc (*, p < 0.05; **, p <0.01; ***, p < 0.001). Ca, capsid; pgRNA, pregenomic RNA; HBc, HBV core protein; WHc, WHV core protein; ssDNA, single-strand DNA; rcDNA, relaxed circular DNA; cccDNA, covalently closed circular DNA; cM-DNA, closed minus strand DNA. (TIF) [file ppat.1010739.s006.tif]

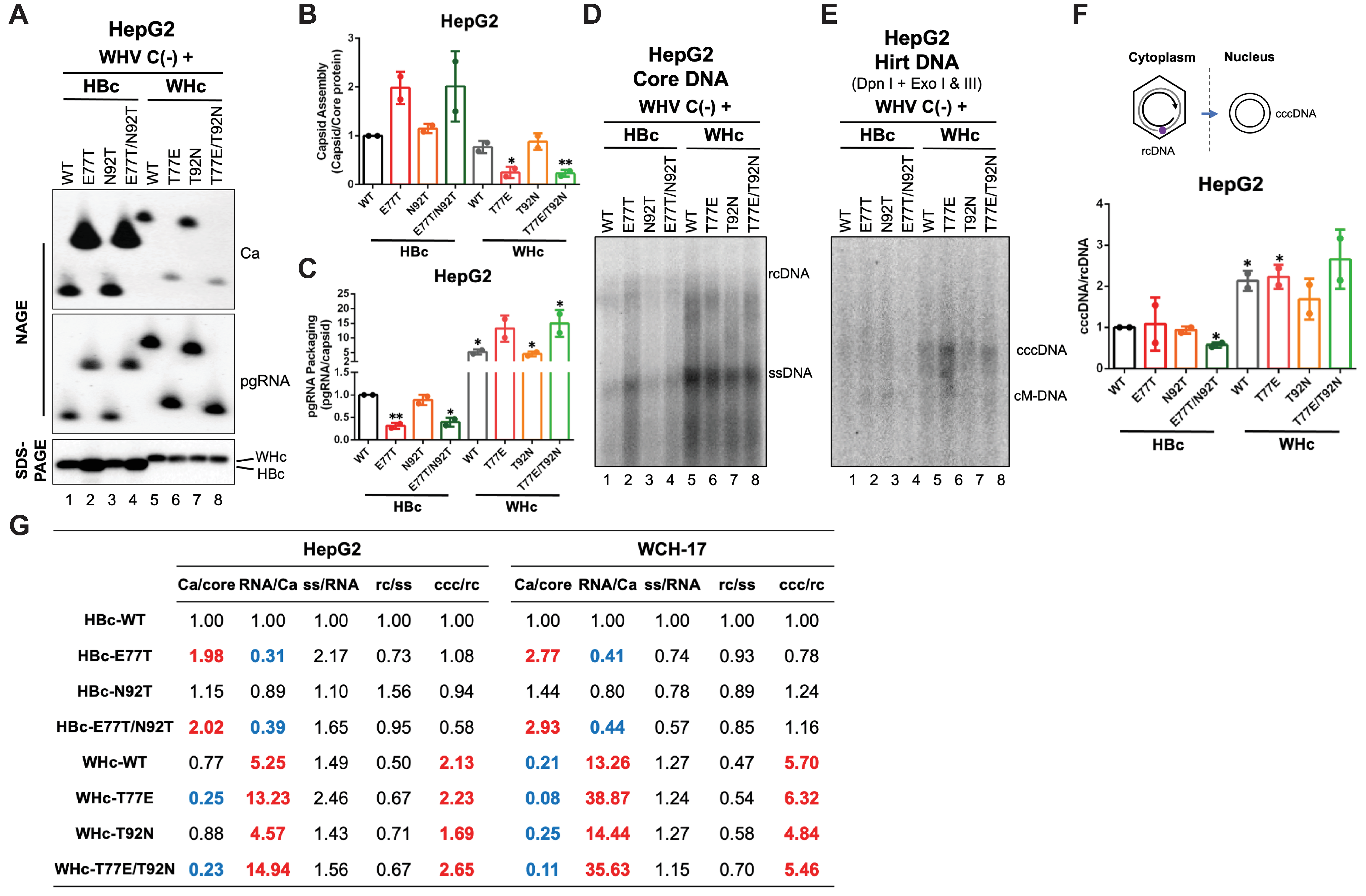

Supplement: S7 Fig — The WHV replicon construct that is defective in WHc expression was co-transfected with WT or mutant HBc or WHc expression construct into HepG2 cells. (A) The assembled capsids (top) and packaged pgRNA (middle) were detected by the C33 anti-HBc/WHc mAb and anti-sense HBV RNA probe, respectively, following the resolution of cytoplasmic lysates by NAGE and transfer to nitrocellulose membrane. Levels of HBc or WHc proteins (bottom) were measured by western blot using 19C18 anti-HBc/WHc mAb after SDS-PAGE. Capsid assembly efficiency (B) was determined by normalizing the levels of capsids to those of total HBc/WHc protein, and pgRNA packaging efficiency (C) was determined by normalizing the levels of pgRNA to those of capsids, with the efficiencies of WT HBc set to 1.0. (D) core DNA was released from the NCs of cytoplasmic lysate by SDS-proteinase K treatment and detected by Southern blot analysis. (E) HBV PF-DNA was isolated from the transfected cells by the Hirt extraction method. The extracted DNA was treated with Dpn I plus Exo I & III to remove all DNA with free 3’ ends. (F) The cccDNA formation efficiency was calculated by normalizing the levels of cccDNA to those of rcDNA, with the efficiency from WT HBc set to 1.0. (G) Summary of all parameters of WHV replication in HepG2 and WCH-17 cells (shown in Fig 6). Increased parameters compared to HBc-WT were marked in red while decreased parameters compared to HBc-WT were marked in blue. Data is shown as mean ± SD. Two-tailed unpaired Student’s t test was used to compare the difference of each data set versus WT HBc (*, p < 0.05; **, p <0.01; ***, p < 0.001). Ca, capsid; pgRNA, pregenomic RNA; HBc, HBV core protein; WHc, WHV core protein; ssDNA, single-strand DNA; rcDNA, relaxed circular DNA; cccDNA, covalently closed circular DNA; cM-DNA, closed minus strand DNA. (TIF) [file ppat.1010739.s007.tif]

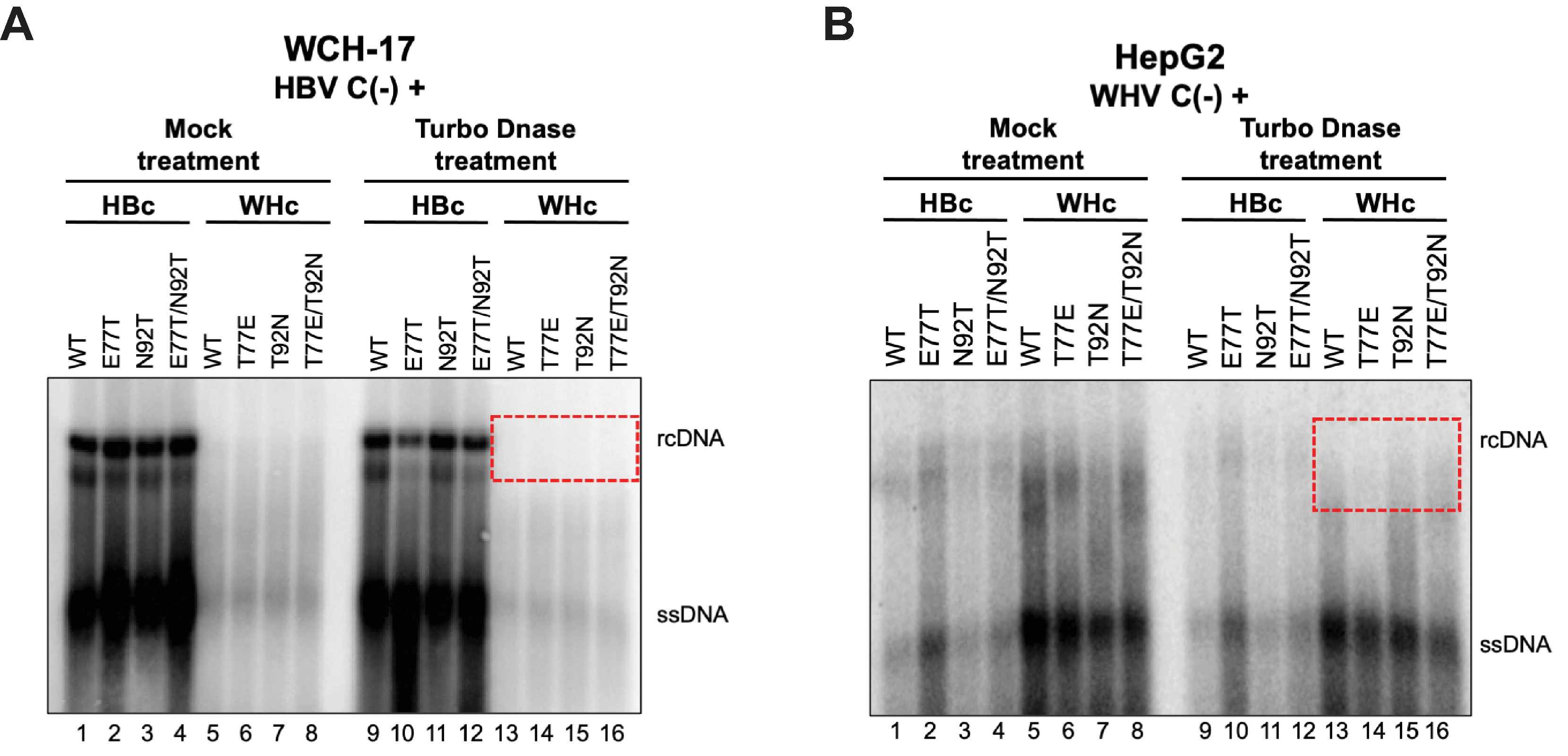

Supplement: S8 Fig — Core DNA released from the cytoplasmic lysate of WT or mutant HBc or WHc co-transfected with HBV-C(-) from WCH-17 cells (A) or with WHV-C(-) from HepG2 cells (B), with or without prior Turbo DNase digestion, was detected by Southern blot analysis. The rcDNA signals obtained after Turbo DNase treatment is indicated by the dashed, red box for comparison to rcDNA signals present without nuclease treatment. ssDNA, single strand DNA; rcDNA, relaxed circular DNA. (TIF) [file ppat.1010739.s008.tif]
